# Transcriptome profiling of low temperature-treated cassava apical shoots showed dynamic responses of tropical plant to cold stress

**DOI:** 10.1186/1471-2164-13-64

**Published:** 2012-02-10

**Authors:** Dong An, Jun Yang, Peng Zhang

**Affiliations:** 1 National Laboratory of Plant Molecular Genetics and National Center for Plant Gene Reserach (Shanghai), Institute of Plant Physiology & Ecology, Shanghai Institutes for Biological Sciences, Chinese Academy of Sciences, 300 Fenglin Road, Shanghai 200032, China; 2 Shanghai Chenshan Plant Science Research Center, Chinese Academy of Sciences, Chenshan Botanical Garden, Shanghai 201602, China

## Abstract

**Background:**

Cassava is an important tropical root crop adapted to a wide range of environmental stimuli such as drought and acid soils. Nevertheless, it is an extremely cold-sensitive tropical species. Thus far, there is limited information about gene regulation and signalling pathways related to the cold stress response in cassava. The development of microarray technology has accelerated the study of global transcription profiling under certain conditions.

**Results:**

A 60-mer oligonucleotide microarray representing 20,840 genes was used to perform transcriptome profiling in apical shoots of cassava subjected to cold at 7°C for 0, 4 and 9 h. A total of 508 transcripts were identified as early cold-responsive genes in which 319 sequences had functional descriptions when aligned with *Arabidopsis *proteins. Gene ontology annotation analysis identified many cold-relevant categories, including 'Response to abiotic and biotic stimulus', 'Response to stress', 'Transcription factor activity', and 'Chloroplast'. Various stress-associated genes with a wide range of biological functions were found, such as signal transduction components (e.g., MAP kinase 4), transcription factors (TFs, e.g., *RAP2.11*), and reactive oxygen species (ROS) scavenging enzymes (e.g., catalase 2), as well as photosynthesis-related genes (e.g., *PsaL*). Seventeen major TF families including many well-studied members (e.g., AP2-EREBP) were also involved in the early response to cold stress. Meanwhile, KEGG pathway analysis uncovered many important pathways, such as 'Plant hormone signal transduction' and 'Starch and sucrose metabolism'. Furthermore, the expression changes of 32 genes under cold and other abiotic stress conditions were validated by real-time RT-PCR. Importantly, most of the tested stress-responsive genes were primarily expressed in mature leaves, stem cambia, and fibrous roots rather than apical buds and young leaves. As a response to cold stress in cassava, an increase in transcripts and enzyme activities of ROS scavenging genes and the accumulation of total soluble sugars (including sucrose and glucose) were also detected.

**Conclusions:**

The dynamic expression changes reflect the integrative controlling and transcriptome regulation of the networks in the cold stress response of cassava. The biological processes involved in the signal perception and physiological response might shed light on the molecular mechanisms related to cold tolerance in tropical plants and provide useful candidate genes for genetic improvement.

## Background

Cassava (*Manihot esculenta *Crantz) is widely cultivated for its starchy storage roots and is a staple food and animal feed in tropical and sub-tropical areas [1]. It is also considered to be an important source of modified starches and bioethanol in China and other Southeast Asian countries [2,3]. Nevertheless, as a tropical root crop, cassava is native to a warm habitat and is categorized as a cold-sensitive species [4]. Thus, low temperatures and frozen conditions are the most important limiting factors for its geographical location and productivity. In the subtropics, where unpredictable cold weather occurs occasionally, it is important to protect the storage roots and propagation stems from chilling stress. For example, the unprecedented freezing disaster occurred in Southern China in January 2008 caused great damage to cassava stem seeds and led to yield reduction in Guangxi, Guangdong and other provinces, resulting in a loss of a billion Chinese Yuan [5]. Furthermore, to ensure a prolonged growth period (i.e., early planting and late harvesting) in the high latitude regions, novel cassava cultivars with improved cold tolerance are in demand.

Under low temperature below 10°C, many species of tropical or subtropical origin are typically injured or killed and show various symptoms of chilling injury due to the inability to adapt to non-freezing low temperatures [6]. For example, cassava exhibits obvious symptoms of damage at these temperatures, including delayed sprouting of the stem cutting, yield decrease, reduced leaf expansion, chlorosis and even necrosis in its leaves [7]. Low temperatures have also been recognized as an important facilitator of decreases in nutrient absorption rates (e.g., Boron), reductions in the leaf photosynthetic rate, and the inhibition of plant growth [4]. In addition, the physiological status of cold-stressed plants is also altered, such as transient increases in hormone levels (e.g., ABA) [8] and changes in membrane lipid composition [9]. Furthermore, the accumulation of compatible osmolytes, such as soluble sugars, betaine, and proline [10-12], and increases in the level of antioxidants [13] are also occurred. In contrast, temperate plants can withstand freezing temperatures following a period of low, but non-freezing, temperatures, a process called cold acclimation [14]. The mechanisms of cold acclimation have been extensively investigated in the model plant *Arabidopsis thaliana *[15], as well as in other important crop species such as maize and barley [16,17]. The most extensively studied mechanism involves a class of ethylene response factors (ERFs) known as the dehydration-responsive element-binding proteins/C-repeat-binding factors (DREBs/CBFs). They interact with the dehydration-responsive element/C-repeat element (DRE/CRT) in the promoters of their downstream target genes to execute a highly coordinated transcriptional response to low temperature signals. Overall, less information is available for tropical plants (e.g., cassava). Therefore, it would be important to increase our understanding of the physiological, biochemical, and molecular characteristics that are associated with cold stress in tropical cassava, especially during the early stages. Moreover, the function of homologous genes in *Arabidopsis *could be used to predict the function of cassava genes which may share the same biological functions or common regulatory scenarios among different plant species [18].

With the development of molecular technologies and 'omics' tools, microarray studies have become a useful strategy for the global analysis of plant gene expression. Using cDNA microarrays or whole genome arrays, the abiotic stress responses of *Arabidopsis *and other plants have been widely analyzed. For examples, the expression patterns of genes were identified in *Arabidopsis *and rice under conditions of drought, cold, high-salinity or abscisic acid treatment [19,20]. The transcriptomic identification of candidate genes involved in sunflower responses to chilling and salt stresses has also been reported [21]. The long-oligonucleotide microarray, which has many advantages compared to other microarray technologies, reliably detects transcript ratios even at one copy per cell in complicated biological samples, and could distinguish different members of gene families [22,23]. Thus, it is appropriate to explore the early cold stress responsive genes of cassava using a 60-mer long-oligonucleotide microarray approach.

Thus far, several studies have been reported in cassava using genomic tools for developing new knowledge and technologies. For instance, EST and cDNA libraries have been constructed in cassava for the identification of starch biosynthesis and biotic/abiotic-responsive genes or genes corresponding to the different developmental stages of various tissues [24-28]. Studies using cassava cDNA microarrays to analyze gene expression in cassava plants subjected to post-harvest physiological deterioration (PPD) or *Xanthomonas axonopodis *infection have also been reported [29,30]. Moreover, gene expression profiling related to the different growth stages of cassava storage roots was recently conducted using a long-oligonucleotide microarray [31]. Fortunately, a draft genome sequence for cassava has been released and recently updated (http://www.phytozome.net/cassava), which greatly facilitates cassava research worldwide. Nevertheless, comprehensive genome-wide expression profiling data for cassava under cold stress treatment are still lacking, which limits our capacity to decipher the molecular mechanisms related to various stress responses. Additionally, to stabilize cassava yield even under unfavorable growth conditions, it is necessary to study the changes of pan-genome genes or pathways in order to identify key candidates for improving cold tolerance in cassava.

In this study, a gene expression profiling of apical shoots from cassava subjected to low temperature was conducted using a custom-designed, 60-mer oligonucleotide microarray covering 20,840 cassava transcripts. In total, 508 differentially expressed genes (DEGs) were identified, and their biological functions were characterized through gene ontology (GO) annotation and KEGG pathway analysis. Our study could increase the understanding of cassava gene regulation under the condition of cold stress and reveal novel approaches to improve cold tolerance through genetic engineering.

## Results and discussion

### Phenotypic and physiological changes of cassava in response to cold

**Figure 1:**
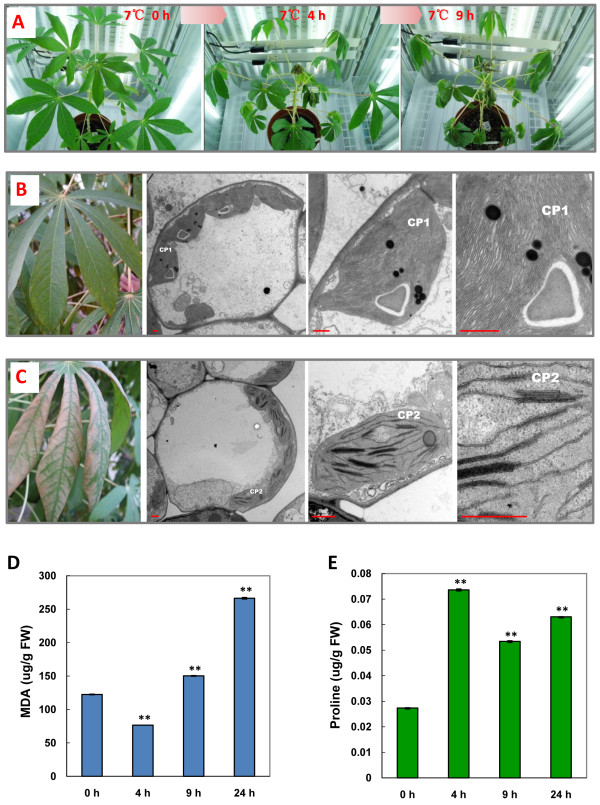
**Phenotypic and physiological changes in cold-stressed cassava**. (**A**) 3-month-old cassava subjected to low temperature stress (7°C) for 0, 4, and 9 h in a chamber under weak light showing phenotypic changes. (**B**) Fully expanded cassava leaf (Left panel) and TEM analysis of a chloroplast (CP1, Right panel). (**C**) Low-temperature treated cassava leaf showing dehydration and necrosis (Left panel) and TEM analysis showing chloroplast abnormalities (CP2, Right panel). (**D**) MDA contents in cassava apical leaves exposed to 7°C for 0, 4, 9, and 24 h. (**E**) Proline accumulation in cassava apical leaves exposed to 7°C for 0, 4, 9, and 24 h. The double asterisks indicate a statistically significant difference (p < 0.01) for the data of the stress-treated samples compared to those of the unstressed samples. The mean values are calculated from three biological replicates; the error bars represent the standard error of the mean (SEM). Bar = 500 nm.

Cassava is a typical tropical crop adapted to warm climates. Under normal conditions, 3-month-old cassava (TMS60444) plants have vigorous apical buds and fully expanded mature leaves with upward leaf fingers (Figure 1A, Left panel). When a low-temperature treatment is applied to cassava at 7°C under weak light for 4 h, the plants displayed visible morphological changes, including weak dehydration and wilting of the apical buds and leaves, especially in the buds, as well as downward leaf fingers (Figure 1A, Middle panel). Under prolonged exposure to the stress (9 h), the whole plants exhibited obvious phenotypic damages, including softening and downward bending of the petioles, loss of strength in the immature stems and more severe wilting leaves (Figure 1A, Right panel). Generally, the treated plants could be partially recovered and resumed growth when transferred into normal conditions (data not shown), indicating that the cellular changes caused by a 9 h cold treatment were reversible. Since apical shoots show extremely sensitive response to low temperature (Additional file 1), the apical shoots (about 4-6 cm) of 3-month-old plants, which were treated for 0, 4 and 9 h, were used as material for microarray hybridization.

In addition to morphological changes, a clear impact on ultra-structure of chloroplasts was also observed in leaves of cold-stressed cassava plants. Compared to normal chloroplasts in unstressed leaves (Figure 1B), the stacking numbers of thylakoids were dramatically reduced and less organized, and the starch granules had almost disappeared (Figure 1C). The ultra-structural changes in cellular organelles indicate that cold stress might exert an adverse impact on plant growth, causing a reduced photosynthetic rate under the stress.

Cold stress also leads to obviously physiological changes in cassava. Two stress responsive metabolites, namely malondialdehyde (MDA) and proline, were monitored. MDA is considered to be the final product of lipid peroxidation in the plant cell membrane and is an important indicator of membrane system injuries and cellular metabolism deterioration [32]. It has also been reported that MDA contents change during cold stress treatment in plants [33]. In our experiment, compared to the control level, the MDA concentrations decreased rapidly to 50% after 4 h but increased almost 25% after 9 h; the observed levels had experienced a nearly two-fold change after 24 h of stress treatment (Figure 1D). Decrease in MDA content at 4 h might be due to a transient stress response of cassava to the low temperature shock. But, the prolonged treatment (9 h) finally led to cell damages and MDA accumulation. The accumulation of proline is frequently associated with whole plant tolerance to chilling and other stresses [34]. Unlike that of MDA, the concentration of proline increased rapidly and achieved nearly a three-fold change after 4 h; it then decreased slightly but remained at a high level until 24 h (Figure 1E). These results were consistent with previous studies that proline accumulated in leaves exposure to cold, salt, and other stresses in *Arabidopsis *and other plants [35]. The accumulation of these metabolites is a good indication that the stress-treated plants were actively mounting a stress response during the periods when they were subjected to cold stress.

### Microarray for transcriptomic analysis of low-temperature treated cassava

**Figure 2:**
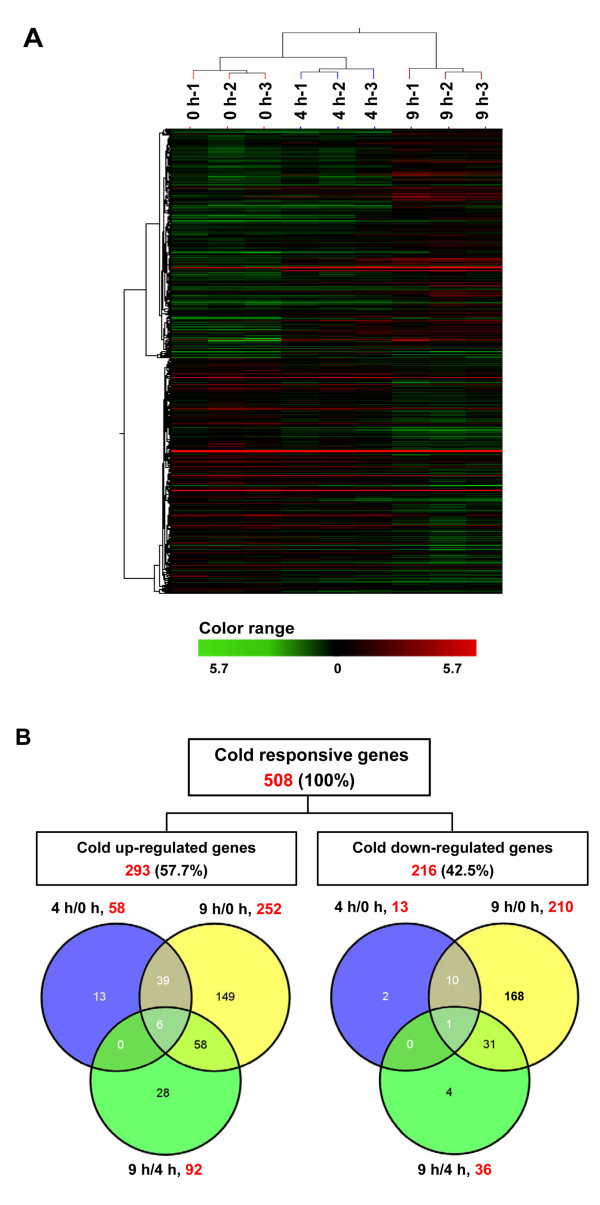
**Expression profiling of cold-regulated genes in cassava apical shoots**. (**A**) Hierarchical cluster analysis. (**B**) Venn diagrams showing cold-regulated genes across three comparisons (4 h/0 h, 9 h/0 h, and 9 h/4 h). The red numbers are the total numbers of differentially expressed genes (DEGs); the percentages in parentheses were calculated as the ratio of regulated genes to the total number of cold-regulated genes (508). It should be noted that one gene was up-regulated at 4 h/0 h but down-regulated at 9 h/4 h on our array.

To explore the transcriptomic changes of cassava in response to cold stress, a custom long-oligonucleotide (60-mer) microarray generated by the Agilent SurePrint ink-jet technology was applied in the current study. The detailed protocol for designing the cassava microarray has been described by Yang et al [31] To validate the microarray quality, the signal-to-noise ratio (SNR) for each spot was calculated as described by Leiske et al [23] The average percentages of acceptable spots (SNR > 2.6) and high-quality spots (SNR > 10) were 94.53 ± 1.84% and 79.24 ± 1.76% in all 9 arrays, which demonstrated the overall reproducibility and high quality of the array. The hierarchical clustering results indicated the clear separation of the samples from different time points (Figure 2A), suggesting that the entire experiment from sample collection to data extraction was reproducible and reliable. Five house-keeping genes (beta-actin, c15, EF1a, RuBisCO small chain precursor, and TUB6) were used as the internal controls and their expressions were consistent in the samples at different time points (Additional file 2), confirming the reliability and accuracy of these microarrays.

### Transcriptomic responses to cold treatment

Before normalization, the signal intensities of each feature were filtered against negative controls on the array, and 69.87 ± 0.72%, 68.14 ± 2.59%, and 71.50 ± 5.75% of probes on the array were expressed at 0, 4 and 9 h, respectively. Two filtering criteria were used to define differentially expressed genes (DEGs) in our data analysis: a two-fold change in transcript levels among every two time points and a *P *value ≤ 0.05. To analyze the similarities and differences among the cold-responsive transcriptomes, a hierarchical clustering was prepared to represent the transcripts of all the differentially expressed probes at the 3 replicates of 0, 4 and 9 h. These results indicated significant differences in the gene expression profiles between the treatments of 9 h and 0 h or 9 h and 4 h, in contrast to the relatively high similarity of the expression profiles between 0 h and 4 h (Figure 2A). Among the DEGs, 58, 252, and 92 genes were up-regulated and 13, 210, and 36 were down-regulated by analyzing 4 h/0 h, 9 h/0 h, and 9 h/4 h, respectively (Figure 2B). Notably, compared to only 71 and 128 genes showed differentially expressed at 4 h/0 h and 9 h/4 h, 474 DEGs were found at 9 h/0 h, suggesting a prolonged stress treatment (9 h) could trigger more stress-related gene expression. Moreover, about 80% of 4 h/0 h and 75% of 9 h/4 h DEGs overlapped with those of 9 h/0 h, which indicated a strong linkage among the 3 stressed comparison points and a progressive biological process.

In total, 508 DEGs, including 292 cold-inducible and 215 cold-repressed genes, were identified on our array; only one gene (CUST_14887) was up-regulated at 4 h/0 h but down-regulated at 9 h/4 h (Additional file 3). These represent about 3.5% of the total expressed cassava transcripts on the array. This result indicated that even for tropical crops (e.g., cassava), which have adapted to warm climates, possess a large amount of cold-responsive genes as other temperate plants (e.g., *Arabidopsis*). Therefore, the differences in the ability of tropical species and temperate species to tolerate cold might not totally be due to the amount of genes responsive to cold stress. Instead, we speculate that plants require to timely and coordinately mobilize the biological functions and regulatory networks of stress-responsive genes against stress. Consistent with our observations, a recent study confirmed such scenario even between two close plant species [36].

### Gene ontology clustering of cold-regulated genes

To explore the biological functions of cold-responsive genes, a total of 20,840 sequences were used as a query to perform an alignment with *Arabidopsis *proteins. Of these, 20,450 sequences had hits with 11,995 *Arabidopsis *proteins, and 14,813 (about 71.8%) sequences had hits with 9,158 *Arabidopsis *proteins at an E value ≤ 1e-5. After searching the descriptions of the 508 DEGs, 319 queries had the highest homologies with annotated proteins according to The *Arabidopsis *Information Resource (TAIR), which accounted for 62.8% of the responsive genes (Additional file 4). To determine the detailed function of the 319 hits with *Arabidopsis *gene locus identifiers, a GO annotation was performed with the GO terms of TAIR (http://www.Arabidopsis.org/tools/bulk/go/index.jsp), in which 370 GO IDs were hit by 291 gene locus identifiers (Additional file 5). Therefore, functional clusters that were classified according to the three components ('Biological Process', 'Molecular Function', and 'Cellular Component') are presented and discussed (Additional files 6, 7, and 8, respectively). Based on the TAIR percent analysis, the categories of 'Response to abiotic and biotic stimulus' and 'Response to stress', 'Transcription factor activity', and 'Chloroplast' were the major cold-related GO Slims that strongly attracted our attention.

#### Cold-responsive genes related to 'Response to abiotic and biotic stimulus' and 'Response to stress'

**Figure 3:**
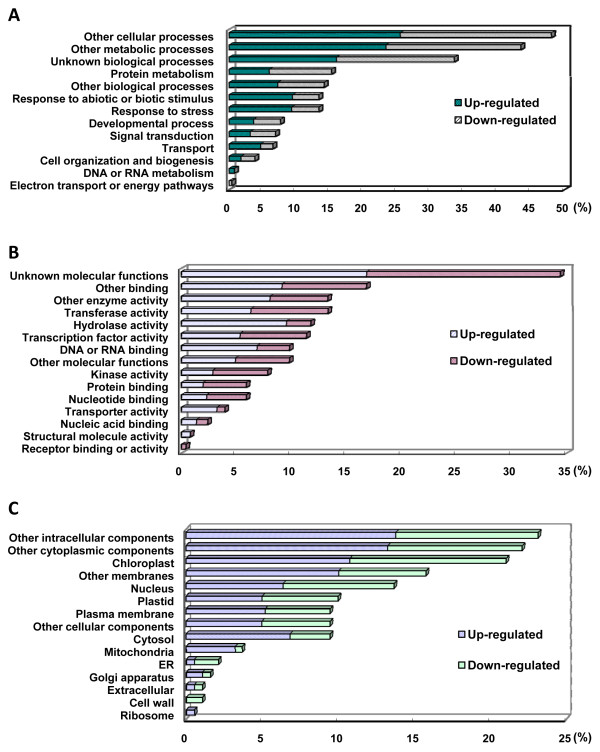
**TAIR percent of gene ontology (GO) terms for (A) 'Biological Process', (B) 'Molecular Function', and (C) 'Cellular Component' of cold up-regulated and down-regulated genes**.

In the GO terms of 'Biological Process', the categories of 'Response to abiotic and biotic stimulus' and 'Response to stress', both accounted for 13.36% of the total GOs, were suggested to be the most relevant to cold stress (Figure 3A). The largest proportions of 'Biological Process' terms were annotated as 'Other cellular processes' (48.09%), 'Other metabolic processes' (43.51%), 'Unknown biological process' (33.59%), and 'Other biological processes' (14.12%), indicating comprehensive changes in cassava gene expression (Figure 3A). In addition, 'Protein metabolism' was another noticeable major category (15.27%) supposed to be also involved in the cold stress response [37].

There were 34 and 33 genes assigned to the GO terms of 'Response to abiotic and biotic stimulus' and 'Response to stress', respectively (Additional file 9). Because 27 genes were common in both categories, we combined the two categories for analysis and discussion (Table 1). Among the overlapping genes, 20 up-regulated genes and 7 down-regulated genes were included. Their encoding proteins cover a wide range of biological functions, including transcription factor (TFs, e.g., AP2 domain transcription factor), protein kinases (e.g., proline extension-like receptor kinase 1), small heat shock proteins (e.g., heat shock protein 18.2), ROS scavenging enzymes [e.g., catalase 2 (*CAT2*)], fatty acid desaturases [e.g., fatty acid biosynthesis 2 (*SSI2*)], signaling molecular proteins (e.g., MAP kinase 4), programmed cell death (PCD)-related gene (e.g., chitinase), and genes involved in the gibberellin acid (GA) and jasmonic acid (JA) metabolism pathway [e.g., gibberellic acid insensitive (*GAI*)]. Among the genes specifically related to the 'Response to abiotic and biotic stimulus', protein degradation genes (e.g., ubiquitin-protein ligase) and early flowering 4 were up-regulated. Meanwhile, genes involved in ABA signal transduction [e.g., ABA-responsive element binding protein 3 (AREB3)] and disease resistance protein (CC-NBS-LRR), which were down-regulated by cold, were uniquely identified in GO Slim as 'Response to stress' (Table 1). Most of these cold-responsive genes were either up-regulated or down-regulated at 9 h/0 h, except 4 genes encoding the calcium ion binding protein (TCH2), ethylene response transcription activator 2 (ERF2), MAP kinase kinase 9, and pseudo-response regulator 5. It is notable that 27 out of these 40 stress-associated genes were up-regulated, indicating that the up-regulation of cold-responsive transcripts by low temperature may play a crucial role in the stress tolerance of plants [38].

**Table 1:** Low temperature responsive transcripts related to 'Response to abiotic and biotic stimulus' and 'Response to stress'

Probe Name	Description	4 h/0 h	9 h/0 h	9 h/4 h	AGI Locus	E Value
				
		Log_2 _Ratio				
**'Response to abiotic and biotic stimulus' and 'Response to stress'**						
CUST_2332	AP2 domain transcription factor	-	4.02	-	AT4G34410	1.60E-23
CUST_14118	SSI2 (fatty acid biosynthesis 2)	2.31	3.69	1.38	AT2G43710	1.80E-57
CUST_12833	alpha-crystallin domain 31.2	-	2.73	2.33	AT1G06460	5.80E-21
CUST_12238	MAP kinase 4	-	2.55	-	AT4G01370	1.30E-16
CUST_5259	proline extensin-like receptor kinase 1	1.18	2.46	1.28	AT3G24550	3.40E-29
CUST_11123	heat shock protein 18.2	-	2.23	1.59	AT5G59720	2.10E-48
CUST_8810	Bet v I allergen family protein	1.81	2.16	-	AT5G28010	4.90E-06
CUST_7667	chitinase	-	2.04	-	AT3G12500	1.50E-11
CUST_11923	MEE14 (maternal effect embryo arrest 14)	-	1.85	1.81	AT2G15890	3.40E-42
CUST_6563	MLP-like protein 31	-	1.72	-	AT1G70840	1.00E-43
CUST_15946	MLP-like protein 31	-	1.42	-	AT1G70840	2.30E-38
CUST_11303	MLP-like protein 34	-	1.42	-	AT1G70850	2.60E-24
CUST_3534	Jasmonic acid carboxyl methyltransferase	-	1.18	-	AT1G19640	5.40E-40
CUST_10998	MLP-like protein 423	-	1.14	-	AT1G24020	2.00E-10
CUST_12659	beta-ketoacyl-CoA synthase	-	1.11	-	AT5G43760	1.40E-63
CUST_3051	CAT2 (catalase 2)	-	1.11	1.01	AT4G35090	7.60E-76
CUST_14725	heat shock protein 17.4	-	1.06	-	AT3G46230	4.10E-33
CUST_17985	TCH2 (calcium ion binding protein)	-	-	1.09	AT5G37770	2.10E-39
CUST_14955	ERF2 (ethylene response transcription activator 2)	-	-	3.35	AT5G47220	1.50E-33
CUST_7141	MAP kinase kinase 9	-	-	1.46	AT1G73500	1.70E-42
CUST_14327	RING-H2 finger A2A zinc ion binding	-	-1.04	-	AT1G15100	5.40E-14
CUST_13235	GAI (gibberellic acid insensitive) transcription factor	-	-1.07	-	AT1G14920	2.00E-108
CUST_14166	GSTU19 (glutathione transferase)	-	-1.12	-	AT1G78380	1.90E-70
CUST_9208	UDP-glucosyltransferase	-	-1.25	-	AT4G01070	8.80E-42
CUST_6759	MYB family transcription factor	-	-1.49	-	AT1G49010	9.30E-41
CUST_5609	RING-H2 finger A2A zinc ion binding	-	-1.97	-	AT1G15100	6.70E-39
CUST_18370	CBL-interacting protein kinase 25	-	-2.03	-	AT5G25110	9.90E-17
**'Response to abiotic and biotic stimulus'**						
CUST_2683	early flowering 4	-	4.01	3.64	AT2G40080	1.00E-25
CUST_4383	gibberellin 2-beta-dioxygenase	-	3.63	-	AT1G30040	4.70E-51
CUST_11786	ubiquitin-protein ligase	-	3.48	3.32	AT1G68050	8.90E-60
CUST_12768	pesudo-response regulator 5	-	-	1.31	AT5G24470	2.80E-20
CUST_14809	SIGB (SIGMA FACTOR B)	-	-1.03	-	AT1G08540	5.30E-20
CUST_14095	polyubiquitin 3 protein binding	-	-1.05	-	AT5G03240	8.90E-17
CUST_11479	zinc ion binding	-	-1.18	-1.01	AT1G52520	1.00E-72
**'Response to stress'**						
CUST_8448	peroxidase 72	-	3.42	2.57	AT5G66390	3.90E-35
CUST_8771	Homolog of yeast autophagy 18	-	2.24	-	AT3G62770	2.80E-63
CUST_11592	ozone-responsive stress-related protein	-	1.72	-	AT1G01170	7.10E-27
CUST_10706	ABA-responsive element binding protein 3	-	-1.18	-	AT3G56850	1.20E-35
CUST_11310	disease resistance protein (CC-NBS-LRR)	-	-1.18	-	AT1G61190	6.40E-06
CUST_18065	ABA-responsive element binding protein 3	-	-1.22	-	AT3G56850	2.30E-20

**Notes: **Low temperature responsive genes are grouped to 'Response to abiotic and biotic stimulus' and 'Response to stress' based on the GO annotation. Positive and negative values of Log_2 _Ratio are either up- or down-regulated genes in the three pair-comparisons. No significant fold changes are indicated by "-".

Of their homologous genes in *Arabidopsis*, the MAP kinase 4, beta-ketoacyl-CoA synthase, *CAT2*, and *TCH2 *were previously reported to be involved in the cold stress response process. For example, MAP kinase 4, which is required for cytokinesis, was implicated in cold and salt stress tolerance [39]; beta-ketoacyl-CoA synthase, which is involved in the biosynthesis of VLCFA (very long chain fatty acids), was also responsive to cold and other osmotic stresses [40]. CAT2, a target of plastid, could increase its transcript abundance in response to cold stress [41]. TCH2 has been implicated in calcium signaling in response to diverse stimuli, such as cold, light, pathogens, and touch [42]. Gibberellin 2-beta-dioxygenase, which degrades active GAs, is involved in cold response [43] and up-regulation of its transcript implied that GA level may be reduced under cold stress. Additionally, calcineurin B-like (CBL)-interacting protein kinase, which has protein serine/threonine kinase activity, has been reported to be involved in the defense response to fungus [44]. These results revealed that there are considerable conserved and common components in cold stress response mechanisms across plant species, including temperate and tropical plants.

Besides, a large number of genes with known function were firstly identified in the cold response. The AP2 domain transcription factor (CUST_2332), a member of the ERF (ethylene response factor) subfamily B-3 of ERF/AP2 transcription factor family, has been reported involved in response to fungus and chitin in *Arabidopsis*. In this study, the transcript abundance of the AP2 domain transcription factor was up-regulated 16-fold by cold, indicating that it may play a significant role in the regulation of novel signaling pathways to enhance cassava cold tolerance. The expression of a stearoyl-ACP desaturase gene *SSI2*, which involves in fatty acid desaturation, was up-regulated almost 10-fold under cold stress. This change suggested that the gene might be participated in altering membrane lipid composition to enhance membrane fluidity. Maternal effect embryo arrest 14 (*MEE14*), which required for embryonic development ending in seed dormancy, was also highly induced in this study. Interestingly, three transcripts (early flowering 4, gibberellin 2-beta-dioxygenase, and pseudo-response regulator 5) that respond to red or far-red light in *Arabidopsis *were cold-inducible, indicating a crosstalk between light signaling and cold stress response.

In addition, a large number of genes with unknown function were also firstly recorded as cold responsive genes. For instance, four members of MLP-like protein (CUST_6563, CUST_15946, CUST_11303, and CUST_10998) were all up-regulated by 9 h cold treatment. Alpha-crystallin domain 31.2, Bet v I allergen family protein, and homology of yeast autophagy 18 were highly induced more than 5-fold by cold. Two members of RING-H2 finger A2A zinc ion binding proteins were also found to be down-regulated. Taken together, cassava might evolve not only conserved but also specific molecular mechanisms related to stress signaling and response.

#### Transcription factors response to cold stress

For the 'Molecular Function' GO terms, 'Transferase activity', 'Hydrolase activity', and 'Transcription factor activity' were the three major categories. They accounted for 13.28%, 11.72%, and 11.33% of the total GOs, respectively (Figure 3B). Among them, genes associated with 'Transcription factor activity' may be central regulators involved in early cold signal transduction that trigger a cascade of downstream gene expression.

Transcription factors (TFs) play a significant role in plant development and stress tolerance [38]. To identify the transcription factors involved in the cold stress response, we surveyed the biological functions of putative TFs that were differentially expressed in cassava under the cold treatment. A total of 32 genes were identified as TFs when the 319 identifiers were annotated in TAIR. The number of up-regulated TFs (15) was nearly equal to the number of down-regulated ones (17), suggesting that both transcriptional activation and repression are involved. The *Arabidopsis *genome has more than 2,657 predicted TFs belonging to 81 gene families in the Plant Transcription Factor Database (PlnTFDB, http://plntfdb.bio.uni-potsdam.de/v3.0/index.php?sp_id=ATH) [45]. According to this classification, 31 cold-responsive transcription factors fell into 17 families, except 1 (CUST_19860) with non-classification (Table 2), and they were annotated with the detailed GO Slims (Additional file 10). In *Arabidopsis*, at least five TF families have been reported to be involved in the cold stress response process, including AP2-EREBP (APETALA2/ET-Responsive Element Binding Protein), MYB (Myeloblastosis), NAC (NAM, ATAF1/2, CUC2), bHLH (basic Helix-Loop-Helix), and WRKY (named after the WRKY amino acid motif) [46].

**Table 2:** Responsive transcription factors during the process of low temperature treatment in cassava apical shoots

Family	Probe Name	Description	4 h/0 h	9 h/0 h	9 h/4 h	AGI Locus	E Value
					
			log_2 _Ratio				
AP2-EREBP	CUST_18605	RAP2.11	2.96	4.59	-	AT5G19790	1.40E-06
	CUST_2332	AP2 domain transcription factor	-	4.02	-	AT4G34410	1.60E-23
	CUST_14955	ERF2 (ethylene response transcription activator)	-	-	3.35	AT5G47220	1.50E-33
	CUST_20765	AP2 domain transcription factor	-	2.02	-	AT1G21910	4.70E-29
	CUST_5161	RAP2.4	-	-	1.15	AT1G78080	8.30E-32
	CUST_2192	ERF9 transcription repressor	-1.51	-	-	AT5G44210	1.00E-21
AUX/IAA	CUST_13515	IAA16 transcription factor	-	-1.55	-1.10	AT3G04730	4.30E-77
ABI3-VP1	CUST_3699	B3 family transcriptional factor	-	-	1.27	AT4G01580	1.80E-17
bZIP	CUST_10706	ABA-responsive element binding protein 3	-	-1.18	-	AT3G56850	1.20E-35
	CUST_18065	ABA-responsive element binding protein 3	-	-1.22	-	AT3G56850	2.30E-20
C2H2	CUST_4530	ZFP5 (ZINC FINGER PROTEIN 5)	-	1.50	-	AT1G10480	9.70E-19
	CUST_12461	C2H2-type zinc finger ZAT-12 like	-	-	2.41	AT2G28710	1.60E-25
C3H	CUST_18218	zinc finger (CCCH-type) family protein	-	-1.05	-	AT4G29190	5.00E-77
G2-like	CUST_13281	PCL1 (PHYTOCLOCK 1)	-	1.62	-	AT3G46640	1.60E-21
	CUST_3593	PCL1 (PHYTOCLOCK 1)	-	1.55	-	AT3G46640	4.90E-35
GRAS	CUST_13235	GAI transcription factor	-	-1.07	-	AT1G14920	2.00E-108
	CUST_7817	scarecrow transcription factor	-	-1.09	-	AT5G66770	1.50E-39
	CUST_12751	SCL5 transcription factor	-	-1.39	-	AT1G50600	2.90E-10
	CUST_10812	scarecrow transcription factor	-	-1.52	-	AT5G66770	8.90E-38
	CUST_6129	scarecrow-like transcription factor 9	-	-	-1.00	AT2G37650	2.90E-15
HSF	CUST_18574	heat shock transcription factor A8	-	2.14	1.74	AT1G67970	1.20E-17
	CUST_14028	heat shock transcription factor C1	-	1.35	1.54	AT3G24520	9.90E-77
JUMONJI	CUST_15293	jumonji domain transcription factor	-	1.85	-	AT4G00990	3.50E-09
LIM	CUST_14929	WLIM1 transcription factor	-	1.21	-	AT1G10200	2.20E-86
MYB	CUST_9065	MYB73	-	-	2.01	AT4G37260	9.60E-25
	CUST_6759	myb family transcription factor	-	-1.49	-	AT1G49010	9.30E-41
MYB-related	CUST_10013	CPC (CAPRICE) transcription factor	-	-1.68	-1.04	AT2G46410	4.10E-12
NAC	CUST_17347	NAC domain transcription factor	-	-1.34	-	AT5G13180	2.60E-14
Sigma70-like	CUST_14809	SIGMA FACTOR B	-	-1.03	-	AT1G08540	5.30E-20
TCP	CUST_7714	PTF1 (PLASTID TRANSCRIPTION FACTOR 1)	-	-1.26	-	AT3G02150	2.00E-12
WRKY	CUST_16901	WRKY7 transcription factor	-	-1.50	-	AT4G24240	2.60E-34
Unclassified	CUST_19860	salt tolerance during germination 1	-1.38	-1.17	-	AT4G31720	5.10E-09

**Notes: **Positive and negative values of Log_2 _Ratio are either up- or down-regulated genes in the three pair-comparisons. No significant fold changes are indicated as "-".

**Figure 4:**
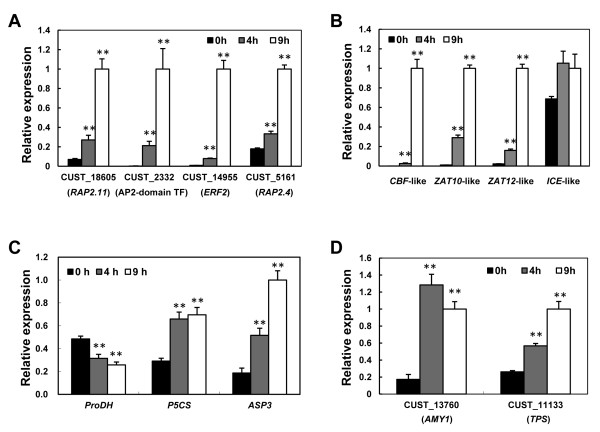
**Expression patterns of important transcripts in response to cold stress at 0, 4, and 9 h by real-time RT-PCR analysis**. (**A**) Members of AP2-EREBP transcription factor family. (**B**) *CBF*-like gene and its upstream regulons. (**C**) *ProDH, P5CS*, and *ASP3 *engaged in 'Proline metabolism'. (**D**) *AMY1 *and *TPS *participated in 'Starch and sucrose metabolism'. The double asterisks indicate a statistically significant difference (p < 0.01) for the data of the stress-treated samples compared to those of the unstressed samples.

In our study, AP2-EREBP, MYB, and GRAS were the three major TF families, containing six, five, and five genes of the 32 cold-responsive TFs, respectively. The AP2-EREBP family plays a major role in the early stages of the cold response, as evidenced by many well-characterized DREBs/CBFs cold regulatory pathway. CBF proteins, belonging to A-1 subfamily of ERF/AP2 TF family, are major regulators that function in activating cold-regulated effectors in *Arabidopsis *and other plants [15]. In our study, all members of AP2-EREBP family are grouped to different subfamilies of ERF/AP2 TF family. For instance, *RAP2.11, ERF2*, AP2 domain transcription factor (CUST_20765), and *RAP2.4 *encode members of B-6, B-3, A-5, and A-6 subfamilies of ERF/AP2 TF family, respectively. Some members have been reported to show response to cold stress in *Arabidopsis*, such as *RAP2.11 *and *RAP2.4 *[47,48]. However, others were firstly identified to be involved in cold response, such as AP2 domain TF (CUST_2332) and *ERF2*. Importantly, most members of this family were highly induced at 9 h during cold treatment, which was confirmed by real-time RT-PCR (Figure 4A), suggesting a relatively delay in triggering transcriptional cascades in the cold response of cassava. Only the ERF9 transcription repressor was down-regulated after 4 h under cold stress. An earlier study had reported that its homologous gene (AT5G44210) of *ERF9 *also experienced a significant decrease in its expression after exposure to heat and freezing in *Arabidopsis *[49]. One of CBF homologous gene (CUST_6694, hereafter *CBF*-like) was found to be up-regulated following 9 h cold treatment. Due to a high *P *value (0.99), it was excluded from the analysis under our standard (*P *value ≤ 0.05). Such scenario indicates that the timely response of DREB/CBF pathway to cold might be variable among different species. Indeed, its cold-induced expression at 9 h following cold treatment, together with most of the AP2-EREBP family factors, was verified by real-time RT-PCR (Figure 4A, B). Therefore, based on our analysis, we speculated that cassava might mount a relatively slow mobilization of stress-related regulators and their downstream genes after exposure to cold, rendering cassava vulnerable to cold stress. Additionally, the transcript of an Inducer of CBF Expression 1 (ICE1)-like gene of cassava was found to be unchanged (Figure 4B). In *Arabidopsis *and other plants, *ICE*s have been reported to be constitutively expressed [50].

A comprehensive expression analysis of MYB TFs demonstrated that almost all MYB TFs are responsive to stresses or hormones [51]. In our study, five MYB or MYB-related family members showed differential expression, with 3 cold-inducible and 2 cold-repressed. Two transcripts, encoding *PCL1 *with a single myb DNA-binding domain, were required for circadian rhythms [52], and the other three MYB/MYB-related transcription factors were responsive to a diverse of hormone treatments in *Arabidopsis *[53]. Differential expression of MYB TFs implies that other environmental or hormonal pathways may be involved in cold response in cassava. Furthermore, the up-regulation of the CUST_3593 transcript (*PCL1*) was also validated by real-time RT-PCR. Members of the GRAS gene family encode transcriptional regulators that have diverse functions in plant growth and development, such as gibberellin signal transduction, root radial patterning, and axillary meristem formation [54]. Although the *Arabidopsis *genome encodes at least 33 GRAS protein family members, few GRAS proteins have been characterized thus far [55]. It is worth noting that all GRAS family members were down-regulated on the array (Table 2), suggesting that cold stress inhibit plant growth and conserve energy to adapt to the adverse environment [56]. Similarly, HSF TFs were reported to be involved in the response to heat and other abiotic stresses [38,57]. The microarray analysis also showed the inducible expression of heat shock transcription factor A8 and C1 following cold treatment (Table 2). Interestingly, A8 was also found to be induced in response to low temperatures in potato and *Arabidopsis *[36]. The induction of heat shock TFs and heat shock proteins (Table 1) revealed that cold and heat stresses may share common responsive elements. The majority of WRKY family TFs is known to be responsive to biotic and/or abiotic stress, despite the fact that most research has focused on the role of these genes in plant-pathogen interactions [58]. WRKY7, known to encode a novel CaM-binding transcription factor of the WRKY group IId in *Arabidopsis *[59], was also found to have differential expression in our study. This might indicate a crosstalk between cold stress response and plant-pathogen interaction in cassava.

In addition to the above-mentioned TFs, we found two AREBs that were cold-repressed in our study. These factors belonged to group A bZIP transcription factors, which play a role in plant pathogen responses, light signaling, and ABA and abiotic stress signaling, and were induced by salt and ABA in *Arabidopsis *[60]. The zinc finger family has been demonstrated to be involved in the cold response in *Arabidopsis *[38]. In our study, we found 3 probes that fell into 2 subfamilies of the zinc finger family (C2H2 and C3H), including 2 up-regulated and 1 down-regulated genes. Zinc finger proteins involve in ROS and abiotic stress signaling in *Arabidopsis *[61]. The C2H2-type zinc finger protein gene *ZAT12*-like was induced not only by cold stress, but also by PEG and salt stress, as illustrated by our real-time RT-PCR results (Figure 4B). This indicates that cassava needs to trigger the expression of ROS-scavenging genes to adapt oxidative stress. The plant-specific NAC transcription factor family has been implicated in plant development processes, such as shoot apical meristem (SAM) maintenance and organ differentiation [62], as well as biotic and abiotic stress responses [63]. We found only one transcript encoding for the NAC domain transcription factor, which was down-regulated on the array. In addition, five novel transcription factor families (ABI3-VP1, JUMONJI, LIM, Sigma70-like, and TCP) were also identified. Their homologous genes in other plant species have not yet been reported in response to cold stress, suggesting that these genes might be specific to cassava and are attractive targets for further functional characterization.

#### Cold-responsive genes related to 'Chloroplast'

The category of 'Chloroplast' was an abundant and important GO Slim. It accounted for 21.06% of total GOs in 'Cellular Component' (Figure 3C, Additional file 11), indicating that a great number of chloroplast-associated genes changed their expression to adapt to cold. This result suggests that the chloroplast might be one of the major organelles affected, as supported by the observation of damaged chloroplasts in cold-stressed leaves (Figure 1C). Although the differential expression of chloroplast-related genes might also be affected by other environmental changes on cassava plants during the treatment, i.e. light change, low temperature was the dominant factor in this study.

Chloroplast is an important organelle unique to plant cells and is the site of photosynthesis. In previous reports, cold stress could reduce the rate of photosynthesis by interfering with the function of many photosynthesis-related proteins [64]. In our study, 43 out of 319 cold-responsive transcripts were assigned to the 'Chloroplast' class, including 22 up-regulated and 21 down-regulated genes (Table 3). Among them, several genes encoding protein localized to thylakoid were identified. For examples, *CAB1 *(chlorophyll a/b binding protein 1), a lipid-associated family protein, a thylakoid leumenal 20 kDa protein, and a hypothetical protein (CUST_7076) were all up-regulated under cold stress. In contrast, *LHCA4 *(photosystem I light harvesting complex gene 4), *PsaL *(photosystem I subunit L), and the rubredoxin family protein were dramatically down-regulated. Two of them (*LHCA4 *and *PsaL*) have also been proposed to be involved in photosynthesis in *Arabidopsis*. These results suggested that differential expression of chloroplast genes, especially those encoding for thylakoid associated proteins, by cold stress might lead to the processes of thylakoid distortion, chloroplast malfunction, and photosynthesis inhibition.

**Table 3:** Low temperature responsive transcripts related to 'Chloroplast'

Probe Name	Description	4 h/0 h	9 h/0 h	9 h/4 h	AGI Locus	E Value
				
		Log_2 _Ratio				
CUST_12882	CCL (CCR-LIKE)	-	4.19	3.26	AT3G26740	1.90E-25
CUST_6863	FAB1 (fatty acid biosynthesis 1)	3.45	4.17	-	AT1G74960	2.10E-13
CUST_9556	CAB1 (chlorophyll a/b binding protein 1)	2.84	4.06	1.22	AT1G29930	9.00E-134
CUST_14118	SSI2 (fatty acid biosynthesis 2)	2.31	3.69	1.38	AT2G43710	1.80E-57
CUST_16917	lipid-associated family protein	2.09	3.48	-	AT4G39730	5.50E-44
CUST_222	PDS1 (PHYTOENE DESATURATION 1)	-	2.47	1.74	AT1G06570	2.00E-13
CUST_19871	dehydrodolichyl diphosphate synthase	-	2.21	-	AT5G58770	1.60E-42
CUST_3426	DNA binding	-	2.15	1.40	AT2G37020	1.60E-44
CUST_11923	MEE14 (maternal effect embryo arrest 14)	-	1.85	1.81	AT2G15890	3.40E-42
CUST_8767	hydrolase, alpha/beta fold family protein	-	1.76	-	AT5G13800	2.70E-33
CUST_4530	ZFP5 (ZINC FINGER PROTEIN 5)	-	1.50	-	AT1G10480	9.70E-19
CUST_15215	thylakoid lumenal 20 kDa protein	-	1.45	1.13	AT3G56650	2.50E-22
CUST_10800	hydrolase	1.03	1.38	-	AT2G35450	4.30E-07
CUST_6415	pentatricopeptide (PPR) repeat-containing protein	-	1.28	-	AT3G02650	9.00E-20
CUST_9148	AMY3 (alpha-amylase-like 3)	-	1.24	-	AT1G69830	2.80E-53
CUST_20805	BIGYIN	1.36	1.22	-	AT3G57090	3.40E-23
CUST_3083	hypothetical protein	-	1.13	-	AT5G59400	1.10E-46
CUST_3051	CAT2 (catalase2)	-	1.11	1.01	AT4G35090	7.60E-76
CUST_4761	hypothetical protein	-	1.07	-	AT4G10000	6.40E-10
CUST_20137	NFU3 (NFU domain protein 3)	-	1.04	-	AT4G25910	4.30E-17
CUST_7076	hypothetical protein	-	1.01	-	AT2G42130	1.30E-40
CUST_4922	CHUP1 (CHLOROPLAST UNUSUAL POSITIONING 1)	-	-1.00	-	AT3G25690	1.60E-09
CUST_14809	SIGB (SIGMA FACTOR B)	-	-1.03	-	AT1G08540	5.30E-20
CUST_14166	GSTU19 (glutathione transferase)	-	-1.12	-	AT1G78380	1.90E-70
CUST_1453	CHUP1 (CHLOROPLAST UNUSUAL POSITIONING 1)	-	-1.13	-	AT3G25690	2.60E-95
CUST_12230	amine oxidase family protein	-	-1.22	-	AT3G09580	4.10E-90
CUST_7714	PTF1 (PLASTID TRANSCRIPTION FACTOR 1)	-	-1.26	-	AT3G02150	2.00E-12
CUST_18664	rubredoxin family protein	-	-1.28	-	AT1G54500	2.90E-43
CUST_1805	hypothetical protein	-	-1.29	-	AT5G40470	3.90E-15
CUST_16156	dehydrodolichyl diphosphate synthase	-	-1.29	-	AT5G58770	1.20E-14
CUST_15952	CHUP1 (chloroplast unusual positioning 1)	-	-1.37	-	AT3G25690	1.70E-69
CUST_19234	LHCA4 (photosystem I light harvesting complex gene 4)	-	-1.39	-	AT3G47470	8.80E-51
CUST_2381	NCED4 (nine-cis-epoxycarotenoid dioxygenase 4)	-	-1.45	-	AT4G19170	1.00E-63
CUST_15061	hypothetical protein	-	-1.48	-1.08	AT3G22970	1.80E-44
CUST_5552	pentatricopeptide (PPR) repeat-containing protein	-	-1.49	-	AT3G42630	1.40E-63
CUST_10020	thioredoxin family protein	-	-1.52	-	AT1G08570	1.40E-43
CUST_2294	ATOFP15/OFP15	-	-1.55	-1.09	AT2G36050	1.40E-28
CUST_7622	DNAJ heat shock protein, putative	-	-1.57	-	AT2G17880	9.20E-25
CUST_5765	DVL13/RTFL2 (ROTUNDIFOLIA LIKE 2)	-	-1.62	-	AT2G29125	2.50E-16
CUST_3996	hypothetical protein	-	-1.64	-1.05	AT1G68430	4.50E-13
CUST_8087	xylulose kinase, putative	-	-1.98	-	AT2G21370	1.60E-56
CUST_6408	PSAL (photosystem I subunit L)	-2.47	-2.31	-	AT4G12800	9.80E-48

**Notes: **Positive and negative values of Log_2 _Ratio are either up- or down-regulated genes in the three pair-comparisons. No significant fold changes are indicated as "-".

### KEGG pathway analysis of cold-responsive genes

**Figure 5:**
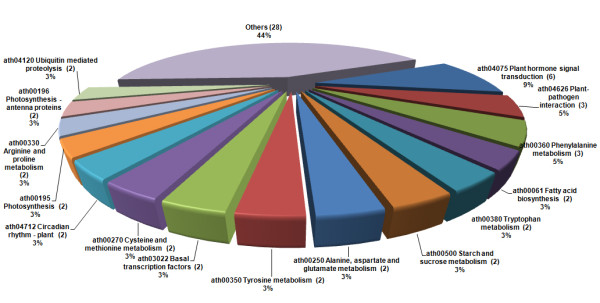
**KEGG pathway maps of cold-responsive genes**. A total of 44 pathways were identified through KEGG mapping. Different colors represent the pathway entries and pathway names. The number of genes represented by AGI loci involved in each pathway is labeled in the parentheses.

To determine whether the cold stress responsive genes engaged in specific pathways, 291 *Arabidopsis *AGI loci representing the 319 DEGs were used as objects to search against KEGG pathway maps in *Arabidopsis thaliana*. Finally, 44 related pathways were identified (Figure 5, Additional file 12). Several interesting and important pathways, including 'Plant hormone signal transduction' (ath04075), 'Plant-pathogen interaction' (ath04626), 'Phenylalanine metabolism' (ath00360), 'Fatty acid biosynthesis' (ath00061), and 'Starch and sucrose metabolism' (ath00500), were involved and function in the early response to cold stress.

'Plant hormone signal transduction' (ath04075) comprised 8 genes of the 44 cold-regulated and pathway-hit genes on our array. In this pathway, the transcripts of *GAI, SAUR *(auxin-responsive protein), *EBF1 *(EIN3-binding F box protein 1), *EIN4 *(ethylene insensitive 4), and *AREB3 *were determined to be down-regulated, and the transcript of *ERF2 *was identified as being up-regulated. Their homologous genes have been reported to be implicated in GA, auxin, ethylene (ETH), and ABA-mediate hormone signal transduction, respectively [65,66]. In plants, these hormones play crucial roles in a diverse set of developmental processes, as well as biotic and abiotic stresses [67]. For example, ABA accumulates in response to abiotic stresses, such as cold, salt, and drought [68]. Among these hormone-related genes in *Arabidopsis, GAI *has been also reported to decrease its transcript levels in response to cold [38], and *ERF2 *has been identified in the response to chitin, a plant-defense elicitor [69]. Previous studies have suggested that the differential regulation of genes involved in hormone signal transduction might play key roles in the early cold stress response [38].

Two other important pathways, including 'Phenylalanine metabolism' (ath00360) and 'Plant-pathogen interaction' (ath04626), were also found in our study to be regulated. *PDS1 *(phytoene desaturation 1), *ASP3 *(Aspartate aminotransferase 3), and peroxidase 72, associated with 'phenylalanine metabolism', were all highly accumulated in response to cold. As noted in previous studies, *PDS1 *has been reported to be engaged in the carotenoid and plastoquinone biosynthetic process [70]. *ASP3*, also participating in proline metabolism, has been shown to be associated with leaf senescence and to be induced in response to darkness and ethylene-induced artificial senescence [71]. In *Arabidopsis *and other plants, proline levels are mainly determined by balance of biosynthetic and catabolic pathways, controlled by *P5CS *(△^1^-pyrroline-5-carboxylate synthetase) and *ProDH *(proline dehydrogenase) genes, respectively [72]. Up-regulation of cassava homologous genes *P5CS *and *ASP3*, and down-regulation of *ProDH*, which were confirmed by real-time RT-PCR (Figure 4C), might correlate with the high accumulation of proline (Figure 1E). Peroxidase 72, a member of the Class III peroxidases, is involved in the response to oxidation stress and potassium resupply [73]. Additionally, transcripts from MAP Kinase 4, *UNE14 *(unfertilized embryo sac 14), and *TCH2*, members of the 'Plant-pathogen interaction' pathway, were also induced by cold (Table 4). MAP Kinase 4 and *TCH2 *have been shown to be involved in response to several stresses, including cold, salinity, and heat in *Arabidopsis *[39]; whereas *UNE14 *also encodes a calcium ion binding protein. These results indicate that calcium ion binding proteins and kinase-mediated signal transduction may play pivotal roles in cold stress response in cassava.

**Table 4:** Validation of selected microarray-based gene expression by real-time RT-PCR analysis

Probe Name	Description	Microarray		qRT-PCR				AGI Locus	Category
		**4 h/0 h**	**9 h/0 h**	**2 h/0 h**	**4 h/0 h**	**9 h/0 h**	**24 h/0 h**		

CUST_9007	JAZ7 (jasmonate-ZIM-domain protein 7)	-	3.92	2.34	**1.96**	3.75	2.82	AT2G34600	UK
CUST_5663	Unknown protein	1.69	3.92	1.20	1.53	2.63	4.19		
CUST_5990	phosphoric monoester hydrolase	-	3.77	0.54	0.11	1.54	2.19	AT1G17710	UK
CUST_4383	gibberellin 2-beta-dioxygenase	-	3.63	5.80	**3.66**	4.02	4.62	AT1G30040	RS
CUST_13673	protein transmembrane transporter	-	3.61	1.41	0.20	1.71	2.08	AT4G16160	UK
CUST_16591	Unknown protein	2.28	3.53	1.41	1.62	1.34	2.61	AT5G37770	RS
CUST_11786	ubiquitin-protein ligase	-	3.48	1.14	**1.15**	1.35	1.91	AT1G68050	RS, CR
CUST_4231	sodium/calcium exchanger protein	-	3.11	0.35	0.60	1.87	1.32	AT1G53210	UK
CUST_20765	AP2 domain transcription factor	-	2.02	0.23	0.42	2.41	1.70	AT1G21910	TF
CUST_12461	C2H2-type zinc finger ZAT-12 like	-	1.97	1.23	**1.39**	1.10	2.57	AT2G28710	TF
CUST_19994	protein phosphatase 2C	-	1.71	0.68	0.39	1.12	0.28	AT3G12620	PM
CUST_3593	PCL1 (PHYTOCLOCK 1)	-	1.55	1.17	0.95	1.96	2.40	AT3G46640	TF
CUST_17985	TCH2 (calcium ion binding protein)	-	1.53	2.66	0.59	1.41	1.79	AT5G37770	PP
CUST_14166	GSTU19 (glutathione transferase)	-	-1.12	-0.82	-0.67	-1.05	-1.00	AT1G78380	RS, CH
CUST_1923	S-adenosylmethionine decarboxylase	-	-1.85	-0.91	**-1.56**	-1.89	-2.63	AT3G02470	AP
CUST_7527	Unknown protein	-1.47	-1.85	-0.43	-1.36	*-***0.92**	-1.06		UK
CUST_19213	Unknown protein	-1.64	-2.59	-0.77	-1.27	-1.28	-1.39		UK
CUST_5741	hypothetical protein	-2.61	-3.05	-1.94	-2.39	-3.04	-3.48	AT1G13360	UK

**Notes: **All data are shown log_2 _Ratio, and positive and negative values of Log_2 _Ratio are either up- or down-regulated genes in the three pair-comparisons. No significant fold changes are indicated as "-". Values of real-time RT-PCR analysis showing in bold font indicate inconsistent data compared with microarray analysis. AP: 'Arginine and proline metablism', CH: 'Chloroplast', CR: 'Circadian rhythm-plant', PM: 'Protein metabolism', PP: 'Plant-pathogen interaction', RS: 'Response to abiotic and biotic stimulus' and 'Response to stress', TF: 'Transcription factor activity', UK: Unknown.

**Figure 6:**
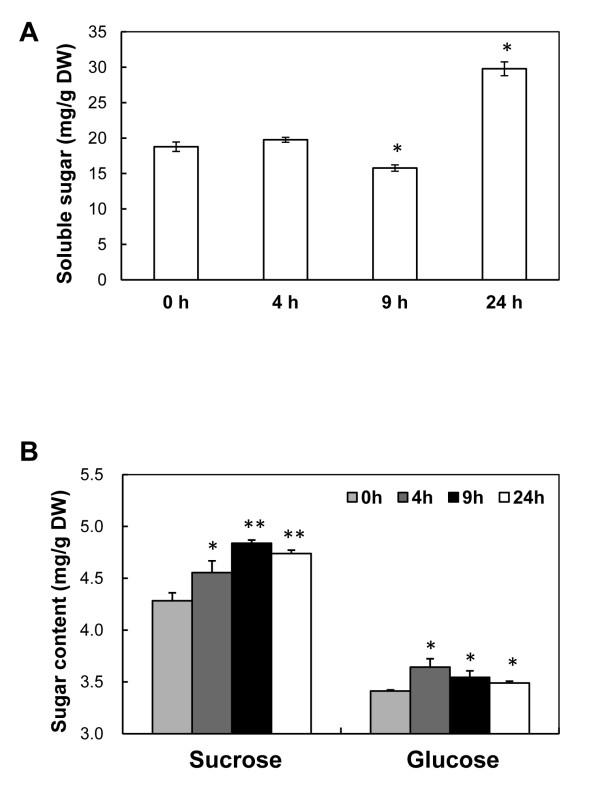
**Contents of total soluble sugars (A) and sucrose and glucose (B) in cassava leaves subjected to cold (7°C) for 0, 4, 9, and 24 h**. The single and double asterisks indicate a statistically significant difference at p < 0.05 and p < 0.01, respectively, for the data of the stress-treated samples compared to those of the unstressed samples. The mean values are calculated from three biological replicates; the error bars represent the standard error of the mean (SEM).

In addition, 'Fatty acid biosynthesis' (ath00061) and 'Starch and sucrose metabolism' (ath00500) were also identified through the pathway analysis. *FAB1 *(fatty acid biosynthesis 1) and *SSI2*, which were also assigned to 'Chloroplast' category, were involved in 'Fatty acid biosynthesis'. Earlier studies in *Arabidopsis *indicated that *FAB1 *is correlated with chilling stress [74], whereas *SSI2 *has been shown to respond to biotic attacks, including viruses, insects, and bacteria [75]. Both were strongly induced during the entire cold treatment process, indicating that membrane modification may occur during the early cold stress response. In *Arabidopsis, TPS7 *[alpha, alpha-trehalose-phosphate synthase (UDP-forming)/transferase] has been reported to participate in the trehalose biosynthetic process. *AMY1 *(alpha-amylase-like 1) has also been shown to take part in the degradation of starch to sugar and to be induced by biotic and abiotic stress [76]. In our study, *TPS7 *and *AMY1 *were identified as being involved in 'Starch and sucrose metabolism'. Their cold-induced expression was confirmed by real-time RT-PCR analysis (Figure 4D). In cold-stressed leaves, starch granules were found to have disappeared (Figure 1C). Even though their total sugar content remained unchanged after 4 h and was lower after 9 h of treatment, an obvious increase was detected after 24 h (Figure 6A). Interestingly, sucrose was significantly accumulated and reached the highest level in 9 h; whereas glucose levels were only slightly increased under cold stress (Figure 6B). These results suggested that soluble sugars might act as both osmolytes and signal molecules in the cold response of cassava, similar to other plant species [77]. It also provided insight into the hypothesis that starch degradation provides conserve energy for plants under adverse conditions. Such transition from synthesis metabolism to degradation metabolism would also explain the reduced productivity observed in plants under stress conditions.

In short, a variety of interesting and important pathways are involved and function in the cellular response to cold stress. Despite many pathways, e.g., 'Plant hormone signal transduction' (ath04075), 'Fatty acid biosynthesis' (ath00061), and 'Starch and sucrose metabolism' (ath00500), have been well-demonstrated in other plant species, regulation of different pathways or gene has been observed, illustrating that a complex and specific network is involved in the early cold response in cassava.

### Validation of microarray results by real-time RT-PCR

To validate our microarray data, real-time RT-PCR analysis was performed on 18 selected differentially expressed transcripts, including 13 up-regulated and 5 down-regulated genes (Table 4). These genes belong to divergent functional categories or pathways. For instance, four genes [gibberellin 2-beta-dioxygenase, unknown protein (CUST_16591), ubiquitin-protein ligase, and *GSTU19*] were involved in 'Response to abiotic and biotic stimulus' and 'Response to stress'. One gene (protein phosphatase 2C) was implicated in 'Protein metabolism,' and other three genes [AP2 domain transcription factor (CUST_20765), C2H2-type zinc finger (*ZAT12*-like), and *PCL1 *(CUST_3593)] were grouped as transcription factors. In addition, 3 genes (ubiquitin-protein ligase, *TCH2*, and S-adenosylmethionine decarboxylase) were assigned to 'Circadian rhythm-plant' (ath04712), 'Plant-pathogen interaction', and 'Arginine and proline metabolism' (ath00330), respectively. Seven genes that are unclassified in the GO Slims or pathways but displayed significant changes in transcript abundance were also tested. For these genes, the fold change (-△△Ct) measured by real-time RT-PCR and by microarray was highly consistent (72.2% and 94.4% for 4 h/0 h and 9 h/0 h, respectively). Their expression kinetics from the real-time RT-PCR results was similar to those of the microarray analysis. These results re-confirmed the accuracy of our microarray data.

### Genes responsive to different stresses and their tissue-specific expressions

The cold stress-signaling pathway may interact with other signaling systems of, for example, ABA, salt, and drought [19]. The expression patterns of the 13 cold-responsive genes were analyzed by real-time RT-PCR using cassava *in vitro *shoot cultures treated with 100 μM ABA, 25% PEG, and 250 mM NaCl for 6 h. Genes with fold changes larger than 4 were defined as strongly responsive to the stresses. Several cold-responsive genes were also strongly induced by ABA, PEG, or salt stress (e.g., CUST_16591, novel protein with unknown function; CUST_12461, C2H2-type zinc finger, *ZAT12*-like; CUST_4383, gibberellin 2-beta-dioxygenase) (Table 5). These results indicated that dynamic crosstalk exists among signaling pathways related to cold, drought, and salt in cassava because all of these stresses cause cellular dehydration [20]. Genes uniquely responsive to cold stress drew more attention in our study, such as *TCH2 *(CUST_17985), AP2 domain transcription factor (CUST_20765), and JAZ7 (jasmonate-ZIM-domain protein 7, CUST_9007) (Table 5). Both TCH2 and JAZ7 were involved in the 'Plant-pathogen interaction' in *Arabidopsis *(Additional file 12) and rice, suggesting that chilling-induced processes share some common features with the defense mechanism against pathogens in plants [58]. In addition, protein transmembrane transporter (CUST_13673), which was strongly induced by ABA and salt stress, was also believed to function in early signal reception.

**Table 5:** Real-time RT-PCR verification of cold responsive gene under the different abiotic stresses and ABA treatment

Probe Name	Description	ABA	PEG	NaCl	Cold	AGI Locus	E Value
				
		Log_2 _Ratio					
CUST_12461	C2H2-type zinc finger ZAT-12 like	1.45	**2.26**	**2.63**	**3.89**	AT2G28710	1.60E-25
CUST_16591	Unknown protein	**2.52**	**4.91**	**2.49**	**2.30**		
CUST_4383	gibberellin 2-beta-dioxygenase	1.12	1.71	**2.52**	**2.65**	AT1G30040	4.70E-51
CUST_17985	TCH2 (calcium ion binding protein)	-0.72	-0.62	-0.41	**2.75**	AT5G37770	2.10E-39
CUST_20765	AP2 domain transcription factor	1.08	-0.61	0.38	**2.16**	AT1G21910	4.70E-29
CUST_9007	JAZ7 (jasmonate-ZIM-domain protein 7)	0.11	-0.57	-0.43	**2.16**	AT2G34600	2.70E-10
CUST_9556	CAB1 (chlorophyll a/b binding protein 1)	1.24	0.21	0.65	1.94	AT1G29930	9.00E-134
CUST_13673	protein transmembrane transporter	**3.22**	1.85	**4.04**	1.73	AT4G16160	3.40E-55
CUST_5990	phosphoric monoester hydrolase	1.21	-1.58	0.09	1.64	AT1G17710	8.60E-90
CUST_14166	GSTU19 (glutathione transferase)	1.00	1.66	1.01	-0.74	AT1G78380	1.90E-70
CUST_19213	Unknown protein	0.41	0.00	-0.13	-1.43		
CUST_7527	Unknown protein	-0.11	-1.21	-1.83	-0.70		
CUST_1923	S-adenosylmethionine decarboxylase	0.59	-0.90	-0.64	-0.70	AT3G02470	1.40E-08

**Notes: **Thirteen selected cold responsive genes were analyzed under different treatments, including 100 μM ABA, 25% PEG, 200 mM NaCl, and cold stress (7°C) for 6 h. Values that were dramatically higher (Log_2 _Ratio > 2) than those of control are shown in bold font.

To further analyze the expression patterns of these stress-regulated genes in different tissues, real-time RT-PCR analysis was conducted. mRNAs were extracted from the apical buds (AP), fibrous roots (FR), mature leaves (ML), stem cambia (SC), young leaves (YL), and young stems (YS) of 3-month-old cassava plants grown in a greenhouse. Interestingly, 7 tested genes were primarily expressed in FR, ML, SC, and YS rather than in AP and YL (Table 6). This tissue-specific expression of cold-responsive genes might explain why the apical shoots, including AP and YL, were more susceptible to cold stress than ML, but this difference remains to be further determined.

**Table 6:** Expression patterns of stress responsive genes in different tissues and organs

Probe Name	Description	AP	FR	ML	SC	YL	YS
		
		Log_2 _Ratio					
CUST_9007	JAZ7 (jasmonate-ZIM-domain protein 7)	1.00	**4.03**	**4.29**	1.48	1.01	**8.05**
CUST_12461	C2H2-type zinc finger ZAT-12 like	1.00	**6.76**	**6.72**	**4.02**	1.93	**5.17**
CUST_5990	phosphoric monoester hydrolase	1.00	**3.32**	**8.24**	**5.32**	0.23	**3.82**
CUST_17985	TCH2 (calcium ion binding protein)	1.00	**3.24**	**3.24**	**2.77**	1.44	**4.57**
CUST_4383	gibberellin 2-beta-dioxygenase	1.00	1.28	1.40	0.88	0.38	**4.78**
CUST_13673	protein transmembrane transporter	1.00	**6.16**	**3.96**	**2.93**	-0.04	1.77
CUST_16591	Unknown protein	1.00	**2.55**	0.74	**3.09**	-0.21	-0.34

**Notes: **AP, apical buds; FR, fibrous roots; ML, mature leaves; SC, stem cambia; YL, young leaves; YS, young stems. All expression data shown were compared with AP. Values that were dramatically higher (Log_2 _Ratio > 2) than those of control are shown in bold font.

### Changes in H_2_O_2 _content and ROS scavenging enzyme activities

**Figure 7:**
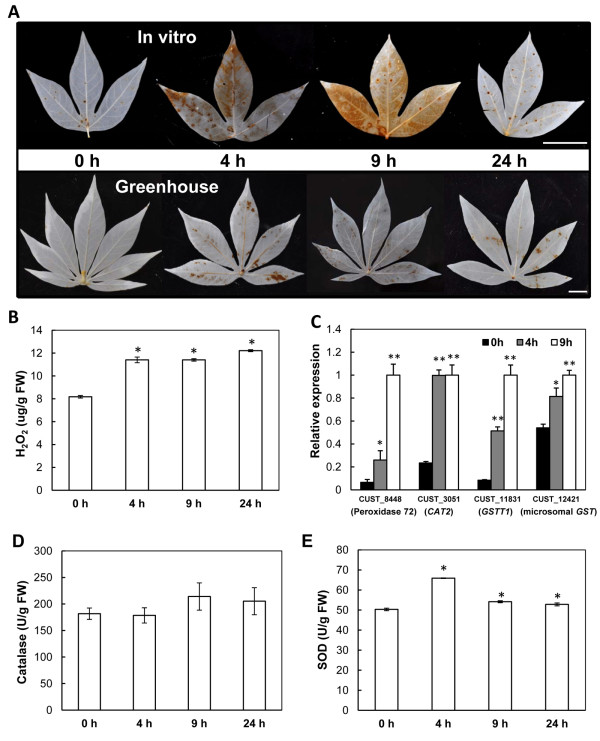
**Detection of H_2_O_2_, transcript level and enzymatic activities of ROS scavenging genes in cassava subjected to cold (7°C) for 0, 4, 9, and 24 h**. (**A**) DAB polymerization of *in vitro *and greenhouse-grown leaves subjected to cold. (**B**) Quantification of H_2_O_2 _content in the leaves from the greenhouse-grown plants. (**C**) The transcript levels of genes encoding for ROS scavenging enzymes in response to cold stress. (**D, E**) Enzyme activities of catalase and superoxide dismutase (SOD) in the leaves of greenhouse-grown plants. The single and double asterisks indicate a statistically significant difference at p < 0.05 and p < 0.01, respectively, for the data of the stress-treated samples compared to those of the unstressed samples. The mean values are calculated from three biological replicates; the error bars represent the standard error of the mean (SEM). Bar = 1 cm.

As previous reported, chilling conditions may lead to an accumulation of ROS such as hydrogen peroxide (H_2_O_2_) and superoxide radical (O_2_^-^) [78]. To analyze and visualize H_2_O_2 _produced in the cassava leaves subjected to cold stress (7°C), diaminobenzidine (DAB) was used to stain the first or second fully expanded leaves of *in vitro *specimens and greenhouse-grown cassava plants. In unstressed leaves, few DAB polymerizations could be observed; in contrast, there was a significant increase in DAB polymerization detected as early as 4 h following cold treatment that continued up to 24 h (Figure 7A). This result was consistent with the quantification of H_2_O_2 _content (Figure 7B), indicating that the oxidative stress may exert a toxic effect on cassava to adapt or survive under cold stress conditions.

Plants, as well as other organisms, have evolved antioxidant systems to protect themselves against toxic species of oxygen. ROS scavenging enzymes, including catalase (CAT), superoxide dismutase (SOD), and glutathione transferase (GST), have been demonstrated to play key roles in the removal of ROS. In our DEGs, several genes encoding ROS scavenging enzymes were recorded to be target to chloroplast, including *CAT2 *and *GSTU19 *(Table 3). In addition, peroxidase 72, and other three *GST*s (*GSTU7, GSTU1*, and microsomal *GST*) were also identified (Additional file 4). The up-regulation of four ROS-scavenging transcripts (CAT2, peroxidase 72, GST1 and microsomal GST) was verified by real-time RT-PCR (Figure 7C). In *Arabidopsis, CAT2 *was reported to be induced by cold stress and *GSTU19 *was responsive to many biotic and abiotic stresses, such as water deprivation and oxidative stress [79]. To further validate the function of ROS scavenging enzymes after exposure to cold stress, the activities of CAT and SOD in cassava leaves subjected to 7°C were measured. Consistent with the microarray data, an increase in CAT activity was observed after 9 h of cold treatment (Figure 7D). Similarly, a significant elevation in SOD activity was also observed after 4 h of cold stress, and such increases continued for 24 h after the initiation of stress treatment (Figure 7E). These results including induction of *ZAT12*-like gene (Figure 4B) suggested that ROS signaling pathways, including ROS scavenging enzymes, were involved in the ROS detoxification induced by the cold stress response in cassava. Similarly, rice and maize, as chilling-sensitive tropical crops, could only withstand transient and milder cold stress. In japonica rice, an oxidative-mediated network has been proposed to play a key role in the early response to chilling stress and short-term defenses [80], and insufficient antioxidant defenses are thought to cause maize chilling sensitivity [81]. Generally, the ultra-structural changes of the chloroplast (Figure 1C) and oxidative burst were typically characteristic of PCD in plants, indicating that PCD may be a part of an adaptive mechanism to survive stress [82].

## Conclusions

**Figure 8:**
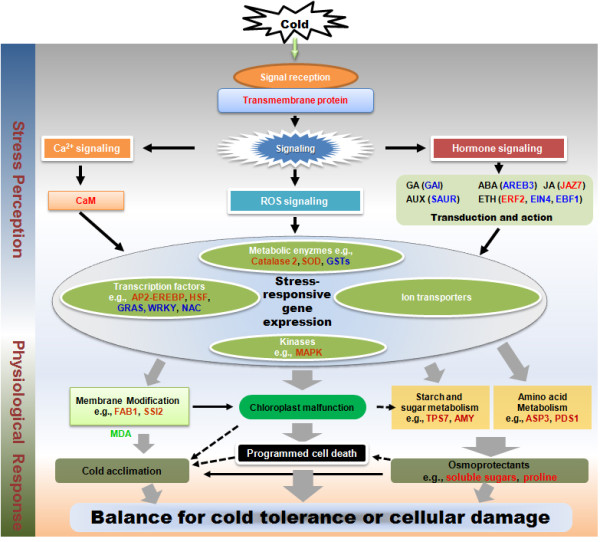
**Molecular model of the early cold response in cassava**. Two biological processes, namely stress perception and the physiological response, are illustrated. After signal reception, stress-activated Ca^2+ ^signaling, ROS signaling, and hormone signaling modulate the expression of stress-responsive genes, which include metabolic enzymes, transcription factors, kinases, and ion transporters. The physiological changes that manifested as membrane modification, chloroplast malfunction, and starch and sucrose metabolism, as well as amino acid metabolism, cause cassava to either have increased cold tolerance or to enter into accelerated programmed cell death. Their balance determines the outcome of the stressed cassava plant. Selected up- and down-regulated genes are in red or blue, respectively. The black arrows indicate a positive effect, and the black dashed arrows indicate a negative effect. The thick gray arrows show different biological processes in the stress response. AMY1: alpha-amylase like 1, AP2-EREBP: APETALA2-Ethylene Responsive Element Binding Protein, AREB3: ABA-responsive element binding protein 3, ASP3: Aspartate aminotransferase 3, AUX: auxin, CaM: calmodulin, EBF1: EIN3-binding F box protein 1, EIN4: ethylene insensitive 4, ERF2: ethylene response transcription activator, ETH: ethylene, FAB1: fatty acid biosynthesis 1, GA: gibberellins, GAI: gibberellic acid insensitive, GRAS: GAI, RGA, and SCR, GSTs: glutathione transferases, HSF: heat shock factor, JA: jasmonate, JAZ7: jasmonate-ZIM-domain protein 7, MAPK: Mitogen-activated protein kinase, PDS1: Phytoene desaturation 1, SAUR: auxin-responsive protein, SOD: superoxide dismutase, SSI2: fatty acid biosynthesis 2, TPS7: trehalose-phosphatase/synthase 7.

Our study presented a genome-wide gene expression profiling of cassava subjected to cold stress using the microarray technology. Overall, the transcriptomic responses of cassava to cold stress were basically consistent with the changes seen in other plants under abiotic stresses [83]. A considerable amount of specific cassava genes related to different biological functions were identified. For examples, many new stress-responsive genes or TFs, such as early flower 4, *ERF2*, and AP2 domain transcription factor (CUST_2332), were found, suggesting that various regulatory pathways may exist in cassava together with the well-characterized CBF pathway. Based on the comprehensive transcriptomic, real-time RT-PCR and physiological analyses, our study supports the fact that crosstalks among different signaling systems play an important role in regulating the cold stress response in tropical plants. Thus, a hypothetical model for depicting the components involved in cold response networks in cassava could be established (Figure 8). Plants may perceive low temperature through cell membrane receptors, most likely transmembrane proteins, and by membrane modification through fatty acid synthesis (e.g., FAB1 and SSI2). Then, the cold signal transduction might induce cellular metabolism changes, such as the calcium signaling cascade, hormone signal transduction, and ROS signaling. The calcium signaling pathway may stimulate the calcium ion binding protein, leading to the activation of cascade kinase activities (e.g., MAP kinase 4), which could switch on various cold stress-responsive genes and transcription factor family proteins (e.g., AP2-EREBP, HSF, and GRAS). Hormone (auxin, ABA, GA, ETH, and JA) signal transduction could also be activated to alter plant growth status in order to adapt to stress condition through the differential regulation of their downstream genes (e.g., SAUR, AREB3, GAI, ERF2, and JAZ7, respectively). Moreover, other metabolisms could also take part in the process, including starch and sucrose metabolism (e.g., TPS7 and AMY1), amino acid metabolism (e.g., ASP3 and PDS1), and the ROS scavenging system (e.g., catalase 2, SOD, and GSTs). Apparently, the balance between cell damage and tolerance might decide the fate for cold-stressed cassava plants (Figure 8). On the other hand, a large number of genes (about 37.2% of total 508 DEGs) encode proteins of unknown functions. Studying these genes may reveal novel mechanisms that are fundamental to the ability of cassava to cope with cold stress.

In summary, our array study will provide the fundamental knowledge related to the biological and physiological changes of cassava under cold stress. It will also be served as a very useful genetic resource for relevant research community globally. Further studies are needed to verify the functions of candidate genes for improving cassava tolerance ability through genetic engineering.

## Methods

### Plant materials used for microarray hybridization

*In vitro *plants of a cassava cultivar (TMS60444) were planted in plastic pots at 28°C under a 16 h light photoperiod (110-150 μmol·m^-2^·s^-1^) for 3 months in the greenhouse. Plants with a uniform growth status were transferred to a chamber for cold treatment at 7°C under weak light (cool-white fluorescent light at approximately 35 μmol·m^-2^·s^-1^). The apical shoots (about 4-6 cm in size, Additional file 1), including apical buds, stems, and the first and second expanded leaves, were harvested after 4 h and 9 h of treatment, then frozen in liquid nitrogen and held at -80°C prior to transporting to Shanghai Biochip Corporation (Shanghai, China) for RNA extraction. Untreated apical shoots were harvested as controls (0 h). More than 3 plants were harvested and pooled for each time point, and the collection was repeated 3 times as biological replicates.

### Physiological analyses of cold-treated cassava plants

To analyze the physiological changes of cassava under cold treatment prior to the microarray study, malondialdehyde (MDA) and proline, indicators of the cold response in plants, were measured. The MDA content was determined by the thiobarbituric acid (TBA) reaction with minor modifications of the method of Dhindsa et al [84] Proline concentrations were measured by the sulfosalicylic acid-acid ninhydrin method according to Bates et al [85] with slight modifications.

### Determination of hydrogen peroxide (H_2_O_2_) content and diaminobenzidine (DAB) staining

Hydrogen peroxide (H_2_O_2_) levels were determined according to Velikova et al [86] The first and second expanded leaves (1 g) were homogenized in an ice bath with 10 mL of 0.1% (w: v) trichloroacetic acid (TCA). The homogenate was centrifuged at 12,000 × *g *for 15 min, and 1 mL of the supernatant was added to 1 mL of 10 mM potassium phosphate buffer (pH7.0) and 2 mL of 1 M KI. The reaction solution containing the supernatant was read at 390 nm using a UV-vis spectrophotometer. The content of H_2_O_2 _was given on a standard curve.

H_2_O_2 _was visualized by staining with DAB, which undergoes a polymerization reaction to yield a dark-brown color once it encounters H_2_O_2 _[87]. The first or second fully developed leaves harvesting from the *in vitro *and greenhouse-grown cassava plants, which were treated with cold stress (7°C) for 0, 4, 9, and 24 h, were excised. Their leaf petiole cuttings were immersed in 1 mg/mL of DAB solution (pH3.8) for 8 h under continuous light. The leaves were immersed in 96% (w/v) boiling ethanol for 10 min to decolorize the chloroplasts. After cooling, the leaves were kept in ethanol and photographed.

### Assay for catalase (CAT) and superoxide dismutase (SOD) activities

For the measurement of enzyme activities, the first and second fully expanded leaves (1 g) from 3-month-old greenhouse plants under cold stress for 0, 4, 9, and 24 h were homogenized in 5 mL of 10 mM potassium phosphate buffer (pH7.0) containing 4% (w: v) polyvinylpyrrolidon (Mr 25 000). The homogenate was centrifuged at 10,000 rpm for 15 min, and the supernatant obtained was used as the enzyme extract. All steps in the preparation of the enzyme extract were carried out at 0-4°C.

CAT activity was determined by directly measuring the decomposition of H_2_O_2 _at 240 nm as described by Cheng et al [88] The reaction mixture contained 50 mM potassium phosphate buffer (pH7.0), 10 mM H_2_O_2_, and 200 μL of enzyme extract in a 2 mL volume. SOD activity assay was based on the method described by Beauchamp and Beaucham et al [89], which measures the inhibition of the photochemical reduction of nitro blue tetrozulium (NBT) at 560 nm. Three milliliters of reaction mixture contained 50 mM phosphate buffer (pH7.8), 0.1 mM EDTA, 13 mM methionine, 75 μM NBT, 16.7 μM riboflavin, and 300 μL of enzyme extract.

### Measurement of total soluble sugars, sucrose and glucose

Soluble sugars were extracted from the leaf tissues by a hot ethanol extraction as follows. Leaves were sampled from 3-month-old cassava, which was treated by cold stress (7°C) at 0, 4, 9, and 24 h and quickly dried at 110°C for 15 min and then 70°C overnight. Exactly 100 mg of homogenized dry leaf powder for each sample was extracted with 8 mL of 80% ethanol (v/v) at 85°C for 40 min. The extracts were then centrifuged at 12,000 × *g *for 10 min. The ethanol extraction step was repeated two times. The 3 resulting supernatants were combined and diluted with 80% ethanol to a volume of 50 mL; they were then treated with 20 mg of activated charcoal at 80°C for 30 minutes. The soluble sugar analysis was conducted according to Ebell [90] with minor modifications. After removing the activated charcoal with a 0.2 μm filter, an aliquot of extraction buffer was reacted with anthrone-sulfuric acid at 95°C for 15 min and then cooled to room temperature. The absorbance of the reaction solution was read at 620 nm. The total soluble sugar content was calculated by a standard curve. Sucrose and glucose content were measured by glucose oxidase method as described by Johnson et al [91] with slightly modification.

### Oligonucleotide microarray preparation, hybridization, and data extraction

The custom-designed 60-mer microarray [31] was constructed based on public sequence information from a large collection of cassava ESTs from NCBI (71,520 ESTs, released 28 March 2008) and TIGR (Manihot_esculenta_release_5, containing 5,189 assemblies and 10,214 singletons, released 1 June 2007), as well as a 35,400 full-length cDNA RIKEN library [27]. Total RNA was extracted from both control and cold-treated tissue samples using an RNeasy Mini Kit (Qiagen, Valencia, CA, USA). The RNA quality was checked on a 1.2% agarose gel using an RNase-free electrophoresis system. RNA labeling and hybridization were conducted by the Shanghai Biochip Corporation (Shanghai, China) following the manufacturer's instructions. The arrays were incubated at 65°C for 17 h in Agilent hybridization chambers (G2545A) and then washed according to the protocol at room temperature. Hybridized microarray slides were scanned at 5 μm resolution with an Agilent Technologies Scanner (G2505B), and the images were saved in JPG format. Both the 10% and 100% Photomultiplier tube (PMT) settings were selected, and the combined images were exported. The signal intensities of all spots on each image were quantified with the Feature Extraction software (Agilent Technologies, Santa Clara, California, USA), and the data were saved as .txt files for further analysis.

### Data normalization and identification of differentially expressed genes (DEGs)

The signal intensity of each gene was globally normalized within the GeneSpring GX Software (Agilent Technologies, Santa Clara, California, USA) following the workflow guide [92]. The signal-to-noise ratio (SNR) was calculated as follows: the difference of the median signal minus the background median signal, divided by the background standard deviation [22]. Pair comparison was used to analyze the normalized and averaged data from the three types of samples (0, 4, and 9 h). The *P *values were calculated using a *t *test, and the fold changes between each comparison for each gene were compared. The genes that were induced or suppressed at levels equal to or greater than a twofold ratio (twofold change cutoff, FCC) were taken to be differentially expressed when SNR > 2.6 and *P *value ≤ 0.05.

### Microarray data analysis

All non-redundant sequences that were considered to be unique cassava genes were locally blasted in the TAIR protein database (27,217 *Arabidopsis *protein sequences), which was download from ftp://ftp.arabidopsis.org/home/TAIR, using the blastx program in the blastall package (version 2.2.9). The top hits were used for gene annotations, and the corresponding *Arabidopsis *gene locus identifiers were mapped to the GO function annotation (http://www.arabiopsis.org/) and to the Kyoto Encyclopedia of Genes and Genomes (KEGG) pathways. The TAIR percent of cold-responsive genes was calculated as follows: The TAIR percent (%) = the number of genes annotated to terms in the GO Slim category divided by N × 100, where N represents the total number of genes from the input list annotated to any term in this ontology.

### Utilization of the microarray

MIAME information about the cassava transcriptome microarray used here has been deposited in the Gene Expression Omnibus (GEO) of NCBI. The accession numbers are: Platform, GPL11271; Series, GSE31073; Samples, GSM769563-GSM769571.

### RNA preparation and real-time RT-PCR

To verify the expression patterns of DEGs identified from the microarray analysis, real-time RT-PCR was conducted using plant materials from either *in vitro *shoot cultures or greenhouse-grown plants. Four-week-old *in vitro *seedlings were transferred into plastic jars containing 150 mL of MS solution [1 × Murashige and Skoog basal salts (MS, Duchefa), 1% sucrose, pH5.7] in a chamber at 25°C, 70% relative humidity, and a light intensity of 125 μmol·m^-2^· s^-1 ^on a 16 h light/8 h dark cycle for 4 days. The solution was then replaced with fresh MS medium (pH5.7) supplemented with 100 μM ABA, 25% PEG, or 200 mM NaCl. Meanwhile, the seedlings were directly transferred to a 7°C chamber for cold stress treatment. After 6 h, whole seedlings were harvested and frozen in liquid nitrogen; they were then stored at -80°C for abiotic stress analysis. Plants in the same hydroponic system with no stress treatment were harvested and used as controls. The apical buds (AP), fibrous roots (FR), mature leaves (ML), stem cambia (SC), young leaves (YL), and young stems (YS) were collected from 3-month-old cassava plants grown in the greenhouse for tissue-specific expression analysis of selected genes. All of the stress treatments had three biological and temporal replicates.

The expression of 32 responsive genes was validated by real-time RT-PCR with RNA samples extracted with a Plant RNA Reagent according to the manufacturer's protocol (Invitrogen, Cat.No.12322-02). The RNA quality was determined by running an agarose gel with ethidium bromide staining. Reverse transcription was performed according to the manufacturer's protocol (TOYOBO, Code: TRT-101). Each cDNA sample was diluted 2 times in sterile ddH_2_O, and 1 μL of this dilution was used as a template for real-time RT-PCR. Specific primers were designed with Primer-BLAST (http://www.ncbi.nlm.nih.gov/tools/primer-blast/) to obtain a T_m _of 60°C and an amplicon length between 70-200 bp (Additional file 13). The real-time RT-PCR reactions were performed in a 20 μL volume containing 10 μL of 2 × SYBR Green Master Mix (TOYOBO, Code: QPK-201), 50 ng cDNA, and 400 nM of forward and reverse primers in a Bio-Rad CFX96 thermocycler. The amplification conditions were as follows: 95°C for 1 min, followed by 40-50 cycles of 95°C for 15 s, 60°C for 20 s, and 72°C for 20 s. A melting curve (65°C-95°C with a heating rate of 0.05°C·s^-1 ^and a continuous fluorescence measurement) was run after the PCR cycles. Beta-actin was used as the internal control. All of the samples were measured in triplicate, and the experiments were performed on three biological replicates. The comparative Ct method was used to calculate the relative gene expression level across the samples. The relative expression level of each gene in one sample (ΔCt) was calculated as follows: Ct target gene-Ct beta-actin. The relative expression of each gene in two different samples (ΔΔCt) was calculated as follows: ΔCt (sample 1) -ΔCt (sample 2).

## Authors' contributions

DA carried out stress treatment and sample collection, microarray data integration, performed real-time RT-PCR experiments, and drafted the manuscript. JY participated in the microarray data analysis and provided helpful suggestions, and PZ was responsible for the overall concept, experimental design, data analysis, and drafting and revising manuscript. All authors read and approved the manuscript.

## Supplementary Material

Additional file 1 **Phenotypic changes of the apical shoots of 3-month-old cassava plants exposed to cold (7°C) at (A) 0, (B) 4, and (C) 9 h**. The apical shoot contains apical bud, young stem, immature leaves, and the first two expanded leaves, as indicated by red box. Bar = 6 cm. Click here for file

Additional file 2 **Stable expression of five internal control genes in the tested samples on the arrays**. beta-actin: *Manihot esculenta *beta-actin; c15: *Manihot esculenta *cytochrome P450 protein CYP71E; EF1a: *Manihot esculenta *elongation factor 1-alpha; RuBisCO: *Manihot esculenta *ribulose bisphosphate carboxylase small chain precursor; TUB6: *Manihot esculenta *Beta-6 Tubulin. Click here for file

Additional file 3 **Differentially expressed genes (DEGs) in the three pair- comparisons from low temperature-treated samples at 0, 4, and 9 h**. Click here for file

Additional file 4 **Gene descriptions of differentially expressed genes based on TAIR protein database**. Excel file contains the 319 DEGs with the highest homologies (AGI locus and full descriptions) and their corresponding E values. Click here for file

Additional file 5 **Gene ontology (GO) annotation of cold responsive genes**. Excel file contains 266 gene locus identifiers with 370 GO IDs that assigned to the three different categories ('Biological Process', 'Molecular Function', and 'Cellular Component'). Click here for file

Additional file 6 **Categories of 'Biological Process' based on GO annotation and their contributing genes**. Click here for file

Additional file 7 **Categories of 'Molecular Function' based on GO annotation and their contributing genes**. Click here for file

Additional file 8 **Categories of 'Cellular Component' based on GO annotation and their contributing genes**. Click here for file

Additional file 9 **GO terms of cold responsive genes related to 'Response to abiotic and biotic stimulus' and 'Response to stress'**. Click here for file

Additional file 10 **GO terms of cold responsive genes belonging to transcription factors**. Click here for file

Additional file 11 **GO terms of cold responsive genes assigned to 'Chloroplast'**. Click here for file

Additional file 12 **KEGG pathway analysis of cold responsive genes**. Excel file contains cold responsive genes with mapped KEGG pathways and their corresponding pathway entries. Click here for file

Additional file 13 **Primers used for real-time RT-PCR verification**. All the forward and reverse primer sequences were included. Click here for file

## References

[B1] El-Sharkawy MA International research on cassava photosynthesis, productivity, eco-physiology, and responses to environmental stresses in the tropics Photosynthetica 2006 44 481 512 10.1007/s11099-006-0063-0

[B2] Cock JH Cassava: a basic energy-source in the tropics Science 1982 218 755 762 10.1126/science.7134971 7134971

[B3] Nguyen TLT Gheewala SH Garivait S Full chain energy analysis of fuel ethanol from cassava in Thailand Environ Sci Technol 2007 41 4135 4142 10.1021/es0620641 17612202

[B4] Huang L Ye Z Bell RW Dell B Boron nutrition and chilling tolerance of warm climate crop species Ann Bot 2005 96 755 767 10.1093/aob/mci228 16033777 PMC4247042

[B5] Chen QL Li Z Fan GZ Zhu KY Zhang W Zhu HQ Indications of stratospheric anomalies in the freezing rain and snow disaster in South China, 2008 Sci China Earth Sci 2011 54 1248 1256 10.1007/s11430-011-4192-3

[B6] Sanghera GS Wani SH Hussain W Singh NB Engineering cold stress tolerance in crop plants Curr Genomics 2011 12 30 43 10.2174/138920211794520178 21886453 PMC3129041

[B7] Alves AAC Hillocks RJ, Thresh JM, Bellotti AC Cassava botany and physiology Cassava: biology, production and utilization CAB Publishing 67 89

[B8] Chandler PM Robertson M Gene-expression regulated by abscisic-acid and its relation to stress tolerance Annu Rev Plant Physiol Plant Mol Biol 1994 45 113 141 10.1146/annurev.pp.45.060194.000553

[B9] Lynch DV Steponkus PL Plasma-membrane lipid alterations associated with cold-acclimation of winter Rye seedlings (Secale-Cereale L-Cv Puma) Plant Physiol 1987 83 761 767 10.1104/pp.83.4.761 16665335 PMC1056446

[B10] Koster KL Lynch DV Solute accumulation and compartmentation during the cold-acclimation of Puma rye Plant Physiol 1992 98 108 113 10.1104/pp.98.1.108 16668599 PMC1080156

[B11] Nomura M Muramoto Y Yasuda S Takabe T Kishitani S The accumulation of glycinebetaine during cold-acclimation in early and late cultivars of barley Euphytica 1995 83 247 250 10.1007/BF01678137

[B12] Dorffling K Dorffling H Lesselich G Luck E Zimmermann C Melz G Jurgens HU Heritable improvement of frost tolerance in winter wheat by *in vitro*-selection of hydroxyproline-resistant proline overproducing mutants Euphytica 1997 93 1 10 10.1023/A:1002946622376

[B13] Pennycooke JC Cox S Stushnoff C Relationship of cold acclimation, total phenolic content and antioxidant capacity with chilling tolerance in petunia (*Petunia *x *hybrida*) Environ Exp Bot 2005 53 225 232 10.1016/j.envexpbot.2004.04.002

[B14] Guy CL Cold-acclimation and freezing stress tolerance: role of protein-metabolism Annu Rev Plant Physiol Plant Mol Biol 1990 41 187 223 10.1146/annurev.pp.41.060190.001155

[B15] Gilmour SJ Zarka DG Stockinger EJ Salazar MP Houghton JM Thomashow MF Low temperature regulation of the Arabidopsis CBF family of AP2 transcriptional activators as an early step in cold-induced COR gene expression Plant J 1998 16 433 442 10.1046/j.1365-313x.1998.00310.x 9881163

[B16] Fernandes J Morrow DJ Casati P Walbot V Distinctive transcriptome responses to adverse environmental conditions in *Zea mays *L Plant Biotechnol J 2008 6 782 798 10.1111/j.1467-7652.2008.00360.x 18643947

[B17] Stanca AM Crosatti C Grossi M Lacerenza NG Rizza F Cattivelli L Molecular adaptation of barley to cold and drought conditions Euphytica 1996 92 215 219 10.1007/BF00022847

[B18] Tatusov RL Koonin EV Lipman DJ A genomic perspective on protein families Science 1997 278 631 637 10.1126/science.278.5338.631 9381173

[B19] Seki M Narusaka M Ishida J Nanjo T Fujita M Oono Y Kamiya A Nakajima M Enju A Sakurai T Satou M Akiyama K Taji T Yamaguchi-Shinozaki K Carninci P Kawai J Hayashizaki Y Shinozaki K Monitoring the expression profiles of 7000 Arabidopsis genes under drought, cold and high-salinity stresses using a full-length cDNA microarray Plant J 2002 31 279 292 10.1046/j.1365-313X.2002.01359.x 12164808

[B20] Rabbani MA Maruyama K Abe H Khan MA Katsura K Ito Y Yoshiwara K Seki M Shinozaki K Yamaguchi-Shinozaki K Monitoring expression profiles of rice genes under cold, drought, and high-salinity stresses and abscisic acid application using cDNA microarray and RNA get-blot analyses Plant Physiol 2003 133 1755 1767 10.1104/pp.103.025742 14645724 PMC300730

[B21] Fernandez P Di-Rienzo J Fernandez L Hopp HE Paniego N Heinz RA Transcriptomic identification of candidate genes involved in sunflower responses to chilling and salt stresses based on cDNA microarray analysis BMC Plant Biol 2008 8 11 10.1186/1471-2229-8-11 18221554 PMC2265713

[B22] Hughes TR Mao M Jones AR Burchard J Marton MJ Shannon KW Lefkowitz SM Ziman M Schelter JM Meyer MR Kobayashi S Davis C Dai H He YD Stephaniants SB Cavet G Walker WL West A Coffey E Shoemaker DD Stoughton R Blanchard AP Friend SH Linsley PS Expression profiling using microarrays fabricated by an ink-jet oligonucleotide synthesizer Nat Biotechnol 2001 19 342 347 10.1038/86730 11283592

[B23] Leiske DL Karimpour-Fard A Hume PS Fairbanks BD Gill RT A comparison of alternative 60-mer probe designs in an in-situ synthesized oligonucleotide microarray BMC Genomics 2006 7 72 10.1186/1471-2164-7-72 16595014 PMC1468409

[B24] Lopez C Jorge V Piegu B Mba C Cortes D Restrepo S Soto M Laudie M Berger C Cooke R Delseny M Tohme J Verdier V A unigene catalogue of 5700 expressed genes in cassava Plant Mol Biol 2004 56 541 554 10.1007/s11103-004-0123-4 15630618

[B25] Anderson JV Delseny M Fregene MA Jorge V Mba C Lopez C Restrepo S Soto M Piegu B Verdier V Cooke R Tohme J Horvath DP An EST resource for cassava and other species of Euphorbiaceae Plant Mol Biol 2004 56 527 539 10.1007/s11103-004-5046-6 15630617

[B26] Lokko Y Anderson JV Rudd S Raji A Horvath D Mikel MA Kim R Liu L Hernandez A Dixon AGO Ingelbrecht IL Characterization of an 18,166 EST dataset for cassava (*Manihot esculenta *Crantz) enriched for drought-responsive genes Plant Cell Rep 2007 26 1605 1618 10.1007/s00299-007-0378-8 17541599

[B27] Sakurai T Plata G Rodriguez-Zapata F Seki M Salcedo A Toyoda A Ishiwata A Tohme J Sakaki Y Shinozaki K Ishitani M Sequencing analysis of 20,000 full-length cDNA clones from cassava reveals lineage specific expansions in gene families related to stress response BMC Plant Biol 2007 7 66 10.1186/1471-2229-7-66 18096061 PMC2245942

[B28] Li YZ Pan YH Sun CB Dong HT Luo XL Wang ZQ Tang JL Chen BS An ordered EST catalogue and gene expression profiles of cassava (*Manihot esculenta*) at key growth stages Plant Mol Biol 2010 74 573 590 10.1007/s11103-010-9698-0 20957510

[B29] Reilly K Bernal D Cortes DF Gomez-Vasquez R Tohme J Beeching JR Towards identifying the full set of genes expressed during cassava post-harvest physiological deterioration Plant Mol Biol 2007 64 187 203 10.1007/s11103-007-9144-0 17318318

[B30] Lopez C Soto M Restrepo S Piegu B Cooke R Delseny M Tohme J Verdier V Gene expression profile in response to *Xanthomonas axonopodis *pv. *manihotis *infection in cassava using a cDNA microarray Plant Mol Biol 2005 57 393 410 10.1007/s11103-004-7819-3 15830129

[B31] Yang J An D Zhang P Expression profiling of cassava storage roots reveals an active process of glycolysis/gluconeogenesis J Integr Plant Biol 2011 53 193 211 10.1111/j.1744-7909.2010.01018.x 21205184

[B32] Hodges DM DeLong JM Forney CF Prange RK Improving the thiobarbituric acid-reactive-substances assay for estimating lipid peroxidation in plant tissues containing anthocyanin and other interfering compounds Planta 1999 207 604 611 10.1007/s004250050524 28456836

[B33] Hodgson RAJ Raison JK Lipid-peroxidation and superoxide-dismutase activity in relation to photoinhibition induced by chilling in moderate light Planta 1991 185 215 219 10.1007/BF00194063 24186344

[B34] Duncan DR Widholm JM Proline accumulation and its implication in cold tolerance of regenerable maize callus Plant Physiol 1987 83 703 708 10.1104/pp.83.3.703 16665311 PMC1056429

[B35] Nanjo T Kobayashi M Yoshiba Y Sanada Y Wada K Tsukaya H Kakubari Y Yamaguchi-Shinozaki K Shinozaki K Biological functions of proline in morphogenesis and osmotolerance revealed in antisense transgenic *Arabidopsis thaliana* Plant J 1999 18 185 193 10.1046/j.1365-313X.1999.00438.x 10363370

[B36] Carvallo MA Pino MT Jeknic Z Zou C Doherty CJ Shiu SH Chen THH Thomashow MF A comparison of the low temperature transcriptomes and CBF regulons of three plant species that differ in freezing tolerance: *Solanum commersonii, Solanum tuberosum*, and *Arabidopsis thaliana* J Exp Bot 2011 62 3807 3819 10.1093/jxb/err066 21511909 PMC3134341

[B37] Howarth CJ Ougham HJ Tansley review .51. Gene-expression under temperature stress New Phytol 1993 125 1 26 10.1111/j.1469-8137.1993.tb03862.x 33874620

[B38] Lee BH Henderson DA Zhu JK The Arabidopsis cold-responsive transcriptome and its regulation by ICE1 Plant Cell 2005 17 3155 3175 10.1105/tpc.105.035568 16214899 PMC1276035

[B39] Teige M Scheikl E Eulgem T Doczi F Ichimura K Shinozaki K Dangl JL Hirt H The MKK2 pathway mediates cold and salt stress signaling in Arabidopsis Mol Cell 2004 15 141 152 10.1016/j.molcel.2004.06.023 15225555

[B40] Joubes J Raffaele S Bourdenx B Garcia C Laroche-Traineau J Moreau P Domergue F Lessire R The VLCFA elongase gene family in *Arabidopsis thaliana*: phylogenetic analysis, 3D modelling and expression profiling Plant Mol Biol 2008 67 547 566 10.1007/s11103-008-9339-z 18465198

[B41] Goulas E Schubert M Kieselbach T Kleczkowski LA Gardestrom P Schroder W Hurry V The chloroplast lumen and stromal proteomes of *Arabidopsis thaliana *show differential sensitivity to short- and long-term exposure to low temperature Plant J 2006 47 720 734 10.1111/j.1365-313X.2006.02821.x 16923014

[B42] Delk NA Johnson KA Chowdhury NI Braam J CML24, regulated in expression by diverse stimuli, encodes a potential Ca^2+ ^sensor that functions in responses to abscisic acid, daylength, and ion stress Plant Physiol 2005 139 240 253 10.1104/pp.105.062612 16113225 PMC1203374

[B43] Thomas SG Phillips AL Hedden P Molecular cloning and functional expression of gibberellin 2-oxidases, multifunctional enzymes involved in gibberellin deactivation Proc Natl Acad Sci USA 1999 96 4698 4703 10.1073/pnas.96.8.4698 10200325 PMC16395

[B44] Huibers RP de Jong M Dekter RW Van den Ackerveken G Disease-specific expression of host genes during downy mildew infection of Arabidopsis Mol Plant Microbe In 2009 22 1104 1115 10.1094/MPMI-22-9-1104 19656045

[B45] Perez-Rodriguez P Riano-Pachon DM Correa LGG Rensing SA Kersten B Mueller-Roeber B PInTFDB: updated content and new features of the plant transcription factor database Nucleic Acids Res 2010 38 822 827 10.1093/nar/gkp1056 19858103 PMC2808933

[B46] Zou CS Yu DQ Analysis of the cold-responsive transcriptome in the mature pollen of Arabidopsis J Plant Biol 2010 53 400 416 10.1007/s12374-010-9129-4

[B47] Ma SS Bohnert HJ Integration of *Arabidopsis thaliana *stress-related transcript profiles, promoter structures, and cell-specific expression Genome Biol 2007 8 10.1186/gb-2007-8-4-r49 PMC1896000 17408486

[B48] Rae L Lao NT Kavanagh TA Regulation of multiple aquaporin genes in Arabidopsis by a pair of recently duplicated DREB transcription factors Planta 2011 234 429 444 10.1007/s00425-011-1414-z 21509693

[B49] Krishnaswamy S Verma S Rahman MH Kav NNV Functional characterization of four APETALA2-family genes (RAP2.6, RAP2.6L, DREB19 and DREB26) in Arabidopsis Plant Mol Biol 2011 75 107 127 10.1007/s11103-010-9711-7 21069430

[B50] Chinnusamy V Ohta M Kanrar S Lee BH Hong XH Agarwal M Zhu JK ICE1: a regulator of cold-induced transcriptome and freezing tolerance in Arabidopsis Gene Dev 2003 17 1043 1054 10.1101/gad.1077503 12672693 PMC196034

[B51] Chen YH Yang XY He K Liu MH Li J Gao ZF Lin ZQ Zhang YF Wang XX Qiu XM Shen YP Zhang L Deng XH Luo J Deng XW Chen ZL Gu HY Qu LJ The MYB transcription factor superfamily of Arabidopsis: expression analysis and phylogenetic comparison with the rice MYB family Plant Mol Biol 2006 60 107 124 10.1007/s11103-005-2910-y 16463103

[B52] Hazen SP Schultz TF Pruneda-Paz JL Borevitz JO Ecker JR Kay SA LUX ARRHYTHMO encodes a Myb domain protein essential for circadian rhythms Proc Natl Acad Sci USA 2005 102 10387 10392 10.1073/pnas.0503029102 16006522 PMC1177380

[B53] Chen YH Yang XY He K Liu MH Li JG Gao ZF Lin ZQ Zhang YF Wang XX Qiu XM Shen YP Zhang L Deng XH Luo JC Deng XW Chen ZL Gu HY Qu LJ The MYB transcription factor superfamily of Arabidopsis: expression analysis and phylogenetic comparison with the rice MYB family Plant Mol Biol 2006 60 107 124 10.1007/s11103-005-2910-y 16463103

[B54] Tian CG Wan P Sun SH Li JY Chen MS Genome-wide analysis of the GRAS gene family in rice and Arabidopsis Plant Mol Biol 2004 54 519 532 15316287 10.1023/B:PLAN.0000038256.89809.57

[B55] Bolle C The role of GRAS proteins in plant signal transduction and development Planta 2004 218 683 692 10.1007/s00425-004-1203-z 14760535

[B56] Achard P Gong F Cheminant S Alioua M Hedden P Genschik P The cold-inducible CBF1 factor-dependent signaling pathway modulates the accumulation of the growth-repressing DELLA proteins via its effect on gibberellin metabolism Plant Cell 2008 20 2117 2129 10.1105/tpc.108.058941 18757556 PMC2553604

[B57] Busch W Wunderlich M Schoffl F Identification of novel heat shock factor-dependent genes and biochemical pathways in *Arabidopsis thaliana* Plant J 2005 41 1 14 15610345 10.1111/j.1365-313X.2004.02272.x

[B58] Dong JX Chen CH Chen ZX Expression profiles of the Arabidopsis WRKY gene superfamily during plant defense response Plant Mol Biol 2003 51 21 37 10.1023/A:1020780022549 12602888

[B59] Park CY Lee JH Yoo JH Moon BC Choi MS Kang YH Lee SM Kim HS Kang KY Chung WS Lim CO Cho MJ WRKY group IId transcription factors interact with calmodulin FEBS Lett 2005 579 1545 1550 10.1016/j.febslet.2005.01.057 15733871

[B60] Choi HI Hong JH Ha JO Kang JY Kim SY ABFs, a family of ABA-responsive element binding factors J Biol Chem 2000 275 1723 1730 10.1074/jbc.275.3.1723 10636868

[B61] Davletova S Schlauch K Coutu J Mittler R The zinc-finger protein Zat12 plays a central role in reactive oxygen and abiotic stress signaling in Arabidopsis Plant Physiol 2005 139 847 856 10.1104/pp.105.068254 16183833 PMC1256000

[B62] Taoka K Yanagimoto Y Daimon Y Hibara K Aida M Tasaka M The NAC domain mediates functional specificity of CUP-SHAPED COTYLEDON proteins Plant J 2004 40 462 473 10.1111/j.1365-313X.2004.02238.x 15500463

[B63] Song SY Chen Y Chen J Dai XY Zhang WH Physiological mechanisms underlying OsNAC5-dependent tolerance of rice plants to abiotic stress Planta 2011 234 331 345 10.1007/s00425-011-1403-2 21448719

[B64] Kingston-Smith AH Harbinson J Williams J Foyer CH Effect of chilling on carbon assimilation, enzyme activation, and photosynthetic electron transport in the absence of photoinhibition in maize leaves Plant Physiol 1997 114 1039 1046 12223758 10.1104/pp.114.3.1039 PMC158392

[B65] Potuschak T Lechner E Parmentier Y Yanagisawa S Grava S Koncz C Genschik P EIN3-dependent regulation of plant ethylene hormone signaling by two Arabidopsis F box proteins: EBF1 and EBF2 Cell 2003 115 679 689 10.1016/S0092-8674(03)00968-1 14675533

[B66] Willige BC Ghosh S Nill C Zourelidou M Dohmann EMN Maier A Schwechheimer C The DELLA domain of GA INSENSITIVE mediates the interaction with the GA INSENSITIVE DWARF1A gibberellin receptor of Arabidopsis Plant Cell 2007 19 1209 1220 10.1105/tpc.107.051441 17416730 PMC1913748

[B67] Jain M Khurana JP Transcript profiling reveals diverse roles of auxin-responsive genes during reproductive development and abiotic stress in rice FEBS J 2009 276 3148 3162 10.1111/j.1742-4658.2009.07033.x 19490115

[B68] Xiong LM Zhu JK Regulation of abscisic acid biosynthesis Plant Physiol 2003 133 29 36 10.1104/pp.103.025395 12970472 PMC523868

[B69] Libault M Wan JR Czechowski T Udvardi M Stacey G Identification of 118 Arabidopsis transcription factor and 30 ubiquitin-ligase genes responding to chitin, a plant-defense elicitor Mol Plant Microbe In 2007 20 900 911 10.1094/MPMI-20-8-0900 17722694

[B70] Norris SR Barrette TR DellaPenna D Genetic dissection of carotenoid synthesis in Arabidopsis defines plastoquinone as an essential component of phytoene desaturation Plant Cell 1995 7 2139 2149 8718624 10.1105/tpc.7.12.2139 PMC161068

[B71] Yoshida S Ito M Nishida I Watanabe A Isolation and RNA gel blot analysis of genes that could serve as potential molecular markers for leaf senescence in *Arabidopsis thaliana* Plant Cell Physiol 2001 42 170 178 10.1093/pcp/pce021 11230571

[B72] Sharma S Villamor JG Verslues PE Essential role of tissue-specific proline synthesis and catabolism in growth and redox balance at low water potential Plant Physiol 2011 157 292 304 10.1104/pp.111.183210 21791601 PMC3165878

[B73] Armengaud P Breitling R Amtmann A The potassium-dependent transcriptome of Arabidopsis reveals a prominent role of jasmonic acid in nutrient signaling Plant Physiol 2004 136 2556 2576 10.1104/pp.104.046482 15347784 PMC523322

[B74] Vijayan P Browse J Photoinhibition in mutants of Arabidopsis deficient in thylakoid unsaturation Plant Physiol 2002 129 876 885 10.1104/pp.004341 12068126 PMC161708

[B75] Chandra-Shekara AC Venugopal SC Barman SR Kachroo A Kachroo P Plastidial fatty acid levels regulate resistance gene-dependent defense signaling in Arabidopsis Proc Natl Acad Sci USA 2007 104 7277 7282 10.1073/pnas.0609259104 17431038 PMC1855359

[B76] Doyle EA Lane AM Sides JM Mudgett MB Monroe JD An alpha-amylase (At4g25000) in Arabidopsis leaves is secreted and induced by biotic and abiotic stress Plant Cell Environ 2007 30 388 398 10.1111/j.1365-3040.2006.01624.x 17324226

[B77] Zhu AD Li WY Ye JL Sun XH Ding YD Cheng YJ Deng XW Microarray expression profiling of postharvest ponkan mandarin (*Citrus reticulata*) fruit under cold storage reveals regulatory gene candidates and implications on soluble sugars metabolism J Integr Plant Biol 2011 53 358 374 10.1111/j.1744-7909.2011.01035.x 21348940

[B78] Suzuki N Mittler R Reactive oxygen species and temperature stresses: A delicate balance between signaling and destruction Physiol Plant 2006 126 45 51 10.1111/j.0031-9317.2005.00582.x

[B79] Bianchi MW Roux C Vartanian N Drought regulation of GST8, encoding the Arabidopsis homologue of ParC/Nt107 glutathione transferase/peroxidase Physiol Plant 2002 116 96 105 10.1034/j.1399-3054.2002.1160112.x 12207667

[B80] Yun KY Park MR Mohanty B Herath V Xu FY Mauleon R Wijaya E Bajic VB Bruskiewich R de los Reyes BG Transcriptional regulatory network triggered by oxidative signals configures the early response mechanisms of japonica rice to chilling stress BMC Plant Biol 2010 10 16 10.1186/1471-2229-10-16 20100339 PMC2826336

[B81] Foyer CH Vanacker H Gomez LD Harbinson J Regulation of photosynthesis and antioxidant metabolism in maize leaves at optimal and chilling temperatures: review Plant Physiol Biochem 2002 40 659 668 10.1016/S0981-9428(02)01425-0

[B82] Koukalova B Kovarik A Fajkus J Siroky J Chromatin fragmentation associated with apoptotic changes in tobacco cells exposed to cold stress FEBS Lett 1997 414 289 292 10.1016/S0014-5793(97)01008-9 9315704

[B83] Sahi C Singh A Blumwald E Grover A Beyond osmolytes and transporters: novel plant salt-stress tolerance-related genes from transcriptional profiling data Physiol Plant 2006 127 1 9 10.1111/j.1399-3054.2005.00610.x

[B84] Dhindsa RS Plumbdhindsa P Thorpe TA Leaf senescence: correlated with increased levels of membrane-permeability and lipid-peroxidation, and decreased levels of superoxide-dismutase and catalase J Exp Bot 1981 32 93 101 10.1093/jxb/32.1.93

[B85] Bates LS Waldren RP Teare ID Rapid determination of free proline for water-stress studies Plant Soil 1973 39 205 207 10.1007/BF00018060

[B86] Velikova V Yordanov I Edreva A Oxidative stress and some antioxidant systems in acid rain-treated bean plants: protective role of exogenous polyamines Plant Sci 2000 151 59 66 10.1016/S0168-9452(99)00197-1

[B87] Thordal-Christensen H Zhang ZG Wei YD Collinge DB Subcellular localization of H_2_O_2 _in plants. H_2_O_2 _accumulation in papillae and hypersensitive response during the barley-powdery mildew interaction Plant J 1997 11 1187 1194 10.1046/j.1365-313X.1997.11061187.x

[B88] Cheng HY Song SQ Possible involvement of reactive oxygen species scavenging enzymes in desiccation sensitivity of antiaris toxicaria seeds and axes J Integr Plant Biol 2008 50 1549 1556 10.1111/j.1744-7909.2008.00723.x 19093973

[B89] Beaucham C Fridovic I Superoxide dismutase: improved assays and an assay applicable to acrylamide gels Anal Biochem 1971 44 276 287 10.1016/0003-2697(71)90370-8 4943714

[B90] Ebell LF Variation in total soluble sugars of conifer tissues with method of analysis Phytochemistry 1969 8 227 233 10.1016/S0031-9422(00)85818-5

[B91] Johnson G Johnson DK Lambert C Sunderwirth SG Colorimetric determination of glucose fructose + sucrose in plant materials using combination of enzymatic + chemical methods J Agr Food Chem 1964 12 216 219 10.1021/jf60133a007

[B92] Bolstad BM Irizarry RA Astrand M Speed TP A comparison of normalization methods for high density oligonucleotide array data based on variance and bias Bioinformatics 2003 19 185 193 10.1093/bioinformatics/19.2.185 12538238

