# Supplementary figures and images for: Transcriptome profiling of low temperature-treated cassava apical shoots showed dynamic responses of tropical plant to cold stress
Source: BMC Genomics. 2012 Feb 10;13:64. doi: 10.1186/1471-2164-13-64 (PMC3339519; doi:10.1186/1471-2164-13-64)

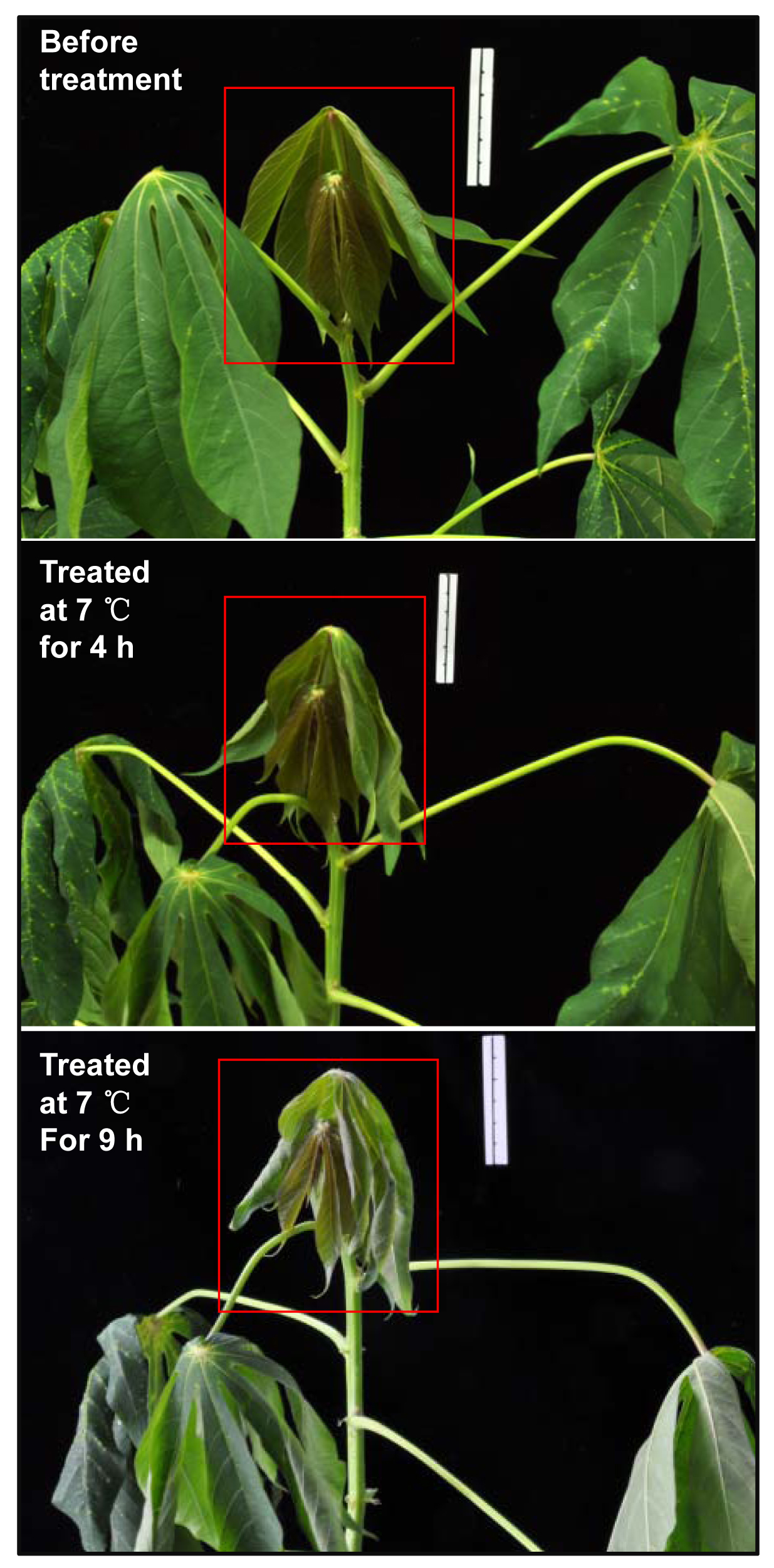

Supplement: Additional file 1 — Phenotypic changes of the apical shoots of 3-month-old cassava plants exposed to cold (7°C) at (A) 0, (B) 4, and (C) 9 h. The apical shoot contains apical bud, young stem, immature leaves, and the first two expanded leaves, as indicated by red box. Bar = 6 cm. [file 1471-2164-13-64-S1.JPEG]

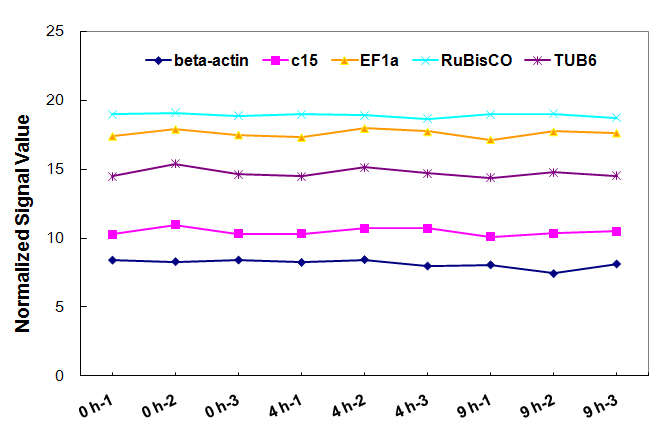

Supplement: Additional file 2 — Stable expression of five internal control genes in the tested samples on the arrays. beta-actin: Manihot esculenta beta-actin; c15: Manihot esculenta cytochrome P450 protein CYP71E; EF1a: Manihot esculenta elongation factor 1-alpha; RuBisCO: Manihot esculenta ribulose bisphosphate carboxylase small chain precursor; TUB6: Manihot esculenta Beta-6 Tubulin. [file 1471-2164-13-64-S2.JPEG]
